# Coronary revascularization and sex differences in cardiovascular mortality after myocardial infarction in 12 high and middle-income European countries

**DOI:** 10.1093/ehjqcco/qcae035

**Published:** 2024-05-07

**Authors:** Edina Cenko, Jinsung Yoon, Maria Bergami, Chris P Gale, Zorana Vasiljevic, Marija Vavlukis, Sasko Kedev, Davor Miličić, Maria Dorobantu, Lina Badimon, Olivia Manfrini, Raffaele Bugiardini

**Affiliations:** Laboratory of Epidemiological and Clinical Cardiology, Department of Medical and Surgical Sciences, University of Bologna, Bologna 40138, Italy; Google Cloud AI, Sunnyvale, 94089 CA, USA; Laboratory of Epidemiological and Clinical Cardiology, Department of Medical and Surgical Sciences, University of Bologna, Bologna 40138, Italy; Clinical and Population Sciences Department, Leeds Institute of Cardiovascular and Metabolic Medicine, University of Leeds, Leeds LS2 9JT, UK; Medical Faculty, University of Belgrade, Belgrade 11000, Serbia; University Clinic for Cardiology, Skopje 1000, Republic of North Macedonia; Faculty of Medicine, Ss. Cyril and Methodius University in Skopje, Skopje 1000, Republic of North Macedonia; University Clinic for Cardiology, Skopje 1000, Republic of North Macedonia; Faculty of Medicine, Ss. Cyril and Methodius University in Skopje, Skopje 1000, Republic of North Macedonia; Department for Cardiovascular Diseases, University Hospital Center Zagreb, University of Zagreb, Zagreb 10000, Croatia; Faculty of Medicine, University of Medicine and Pharmacy “Carol Davila”, Bucharest 014461, Romania; Cardiovascular Research Program ICCC, IR-IIB Sant Pau, Hospital de la Santa Creu i Sant Pau, CiberCV-Institute Carlos III, Barcelona 08025, Spain; Laboratory of Epidemiological and Clinical Cardiology, Department of Medical and Surgical Sciences, University of Bologna, Bologna 40138, Italy; Cardiology Unit, IRCCS Azienda Ospedaliero-Universitaria di Bologna Sant'Orsola Hospital, Bologna 40138, Italy; Laboratory of Epidemiological and Clinical Cardiology, Department of Medical and Surgical Sciences, University of Bologna, Bologna 40138, Italy

**Keywords:** Sex differences, Myocardial infarction, Outcomes, Middle-income countries

## Abstract

**Background:**

Existing data on female sex and excess cardiovascular mortality after myocardial infarction (MI) mostly come from high-income countries (HICs). This study aimed to investigate how sex disparities in treatments and outcomes vary across countries with different income levels.

**Methods:**

Data from the ISACS Archives registry included 22 087 MI patients from 6 HICs and 6 middle-income countries (MICs). MI data were disaggregated by clinical presentation: ST-segment elevation myocardial infarction (STEMI) and non-ST-segment elevation myocardial infarction (NSTEMI). The primary outcome was 30-day mortality.

**Results:**

Among STEMI patients, women in MICs had nearly double the 30-day mortality rate of men [12.4% vs. 5.8%; adjusted risk ratio (RR) 2.30, 95% CI 1.98–2.68]. This difference was less pronounced in HICs (6.8% vs. 5.1%; RR 1.36, 95% CI 1.05–1.75). Despite more frequent treatments and timely revascularization in MICs, sex-based mortality differences persisted even after revascularization (8.0% vs. 4.1%; RR 2.05, 95% CI, 1.68–2.50 in MICs and 5.6% vs. 2.6%; RR 2.17, 95% CI, 1.48–3.18) in HICs. Additionally, women from MICs had higher diabetes rates compared to HICs (31.8% vs. 25.1%, standardized difference = 0.15). NSTEMI outcomes were relatively similar between sexes and income groups.

**Conclusions:**

Sex disparities in mortality rates following STEMI are more pronounced in MICs compared to HICs. These disparities cannot be solely attributed to sex-related inequities in revascularization. Variations in mortality may also be influenced by sex differences in socioeconomic factors and baseline comorbidities.

## Graphical Abstract

**Graphical Abstract:**
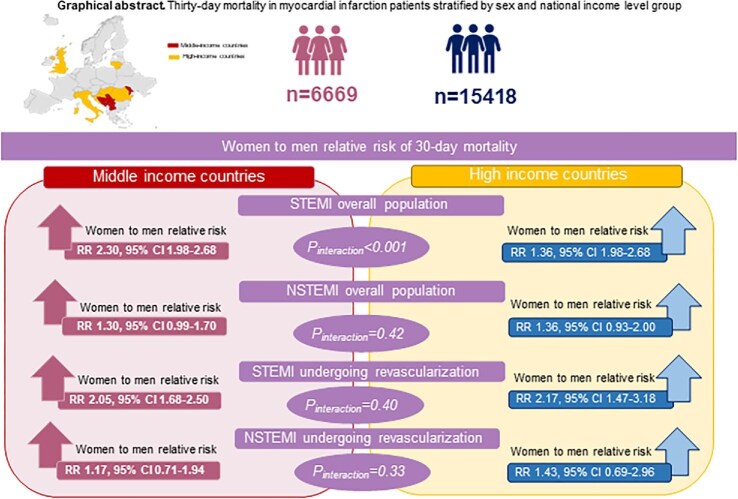

Key Learning Points What is already known

- A significant portion of the available data on the relationship between female sex and cardiovascular mortality from MI comes from European high-income countries (HICs).
- We did not find any comprehensive report on differences between women and men in risk factors and post-MI outcomes in European middle-income countries (MICs), where there are often lower healthcare resources.

What this study adds

- Among patients with STEMI, the proportion of women who died within 30 days was around twice more than men in MICs, even after extensive adjustment for confounding variables. This difference was much lower in HICs.
- The persistence of higher absolute mortality rates in women from MICs compared with HICs after timely coronary revascularization and the greater rates of diabetes among women in MICs underscore the importance of considering baseline risk factors for achieving more equitable outcomes, regardless of their economic context.
- Implementing primary prevention programs is crucial in European MICs and may result in a viable opportunity to reduce the sex gap in mortality.

## Introduction

Most available data on the relationship between female sex and cardiovascular mortality from myocardial infarction (MI) are from countries with the highest incomes, where there are often better healthcare resources. However, cardiovascular disease is a leading cause of mortality worldwide, and understanding its patterns in diverse populations is crucial for developing effective prevention and treatment strategies. In this context, recent population-based studies have suggested that revascularization procedures for MI were less frequently used in women than men, but this was paradoxically associated with no sex differences in outcome rates in women compared with men.^1,2^ These unexpected results have raised uncertainty about the value of existing quality measures of care and questions about the true relation between coronary revascularization and mortality from MI in women. Confirmation of such an assertion needs to be supported by concurrent data on the sex-specific individual risk profile and severity of clinical presentation of the population that receives revascularization therapy.

To address these questions, we analysed the European Cardiovascular Disease Statistics Database and the International Survey of Acute Coronary Syndromes (ISACS) Archives registry involving a large cohort of patients who presented with MI involving 12 European countries: 6 high-income countries (HICs) and 6 middle-income countries (MICs). The main goal of our study was to determine the association of country’s income level with sex differences in outcomes after MI. This goal was achieved by comparing groups of patients with equal severity of the disease, specifically with ST-segment elevation myocardial infarction (STEMI) and non-ST-segment elevation myocardial infarction (NSTEMI). A further aim of the study was to unravel the relation between revascularization and mortality from MI among women and men in MICs and HICs. Our hypothesis was that the baseline clinical characteristics of patients in countries with different income levels may contribute to different absolute rates of mortality after revascularization.

## Methods

### Study design and setting

We analysed information from the ISACS Archives (NCT04008173) from October 2010 to January 2021. We identified two large clinical registries providing information on MI and its relationship with early death, specifically the ISACS-TC (NCT01218776) and the EMMACE-3X (Long-term Follow-up of Health-Related Quality of Life in Patients with Acute Coronary Syndrome; NCT01955525). Details of the study design, sampling, and recruitment have been previously published and are also described in the Supplementary material online. In brief, the ISACS-TC registry collected data from 40 centres in 12 European countries: Bosnia and Herzegovina, Croatia, Italy, Kosovo, Lithuania, Hungary, North Macedonia, Moldova, Montenegro, Romania, Serbia, and the United Kingdom. Among these sites, there are 22 tertiary health care services providing percutaneous coronary intervention (PCI).^3^ The EMMACE-3X gathered routine clinical information from 47 hospitals in England. Cardiovascular facilities including PCI were available in 33 hospitals.^4^ This study complies with the Declaration of Helsinki. The local research ethics committee from each hospital approved the study. Because patient information was collected anonymously, institutional review boards waived the need for individual-informed consent. Both registries had independent source documentation. All data were transferred to the Department of Electrical and Computer Engineering, University of California, Los Angeles, where final statistical analyses were done.

### Participants

**Figure 1:**
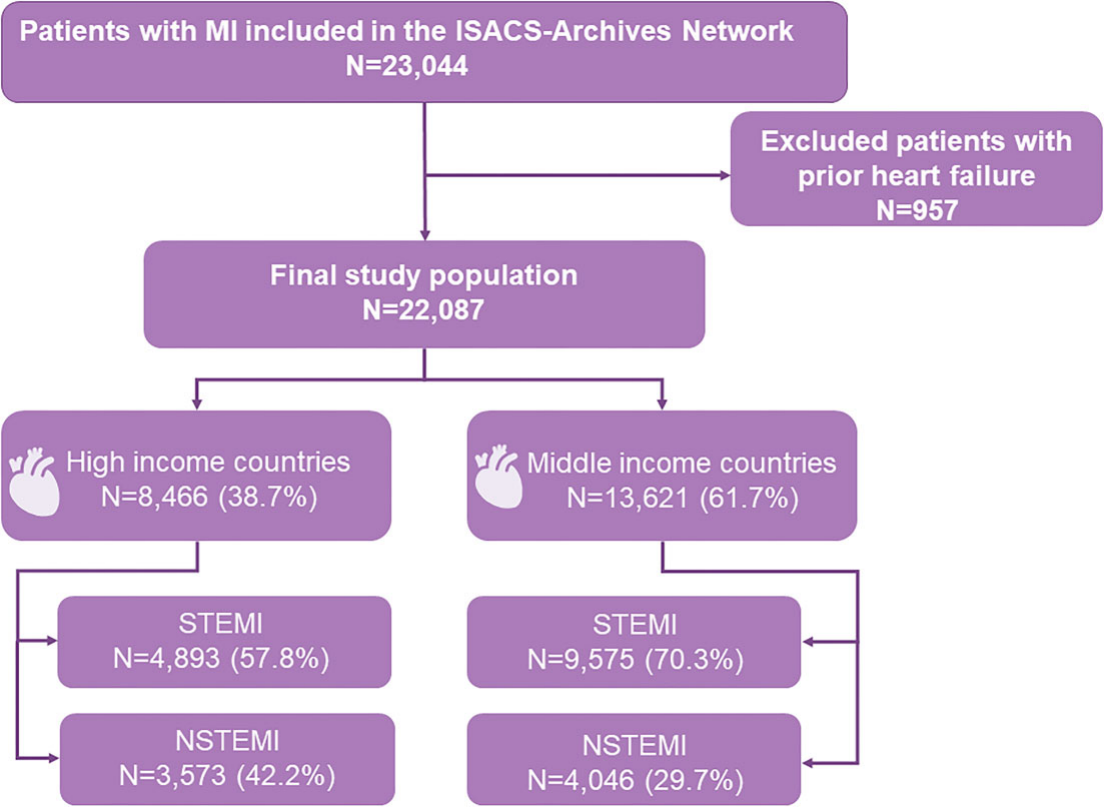
Flow chart of the study cohort. Patient inclusion and exclusion criteria from the ISACS Archives registry. Patients were considered eligible if they had clinically confirmed myocardial infarction. The initial group consisted of 23 044 patients. Patients presenting heart failure were excluded leaving a final study group of 22 087 patients. MI, myocardial infarction; NSTEMI, non-ST-segment elevation myocardial infarction; STEMI, ST-segment elevation myocardial infarction.

The study population consisted of 22 087 patients with MI (*Figure 1*). The designated physician collected the registry data at the time of clinical assessment. The diagnosis was validated by two cardiologists based on the presence of symptoms plus electrocardiogram changes and biomarker release indicative of MI.^5^

### World health organization and European heart network

Using the World Bank Atlas classification 2021 of income levels, we analysed six MIC with a gross national annual income (GNI) per capita between ${\$}$1136 and ${\$}$13 845 (Bosnia and Herzegovina, Kosovo, North Macedonia, Moldova, Montenegro, and Serbia) and 6 HIC with a GNI per capita of ${\$}$13 846 or more (Croatia, Italy, Lithuania, Hungary, Romania, and the United Kingdom).^6^

### European cardiovascular disease statistics database

Ischaemic heart disease (IHD) age-standardized prevalence and mortality from IHD data came from the European Cardiovascular Disease Statistics Database, using the 2017 or latest year available.^7^ To examine whether the association between income status and sex differences in outcome may change, we compared the women-to-men rate ratios for death from IHD in relation to the prevalence of IHD per 100 000 inhabitants. To calculate the rate ratio, first we calculated the death rate per 100 000 inhabitants among the prevalence rate per 100 000 inhabitants for each group. Next, the rate for the group of primary interest (women) was divided by the rate for the comparison group (men). A rate ratio of 1.0 indicates equal rates in the two groups; a rate ratio greater than 1.0 indicates a relative increased risk for women; and a rate ratio less than 1.0 indicates a relative decreased risk for women.

### Outcome measures and definitions

The primary outcome measure was 30-day all-cause mortality from hospital admission. The 30-day window was selected to enrich the data over that acquired during the index hospitalization while mitigating survivor bias. We noted the types of evidence-based medications [aspirin, P2Y_12_ inhibitors, statins, angiotensin-converting enzyme (ACE)-inhibitors, angiotensin receptor blockers (ARB), β-blockers] given prior to the index event. We also noted the use of revascularization and the types of medications given on hospital admission (fibrinolytic agents, aspirin, and P2Y_12_ inhibitors) and on discharge (aspirin, clopidogrel, β-blockers, statins, and ACE-inhibitors/ARBs). In the current analyses, revascularization strategy was defined as the use of PCI. Coronary artery bypass graft (CABG) procedures were performed only as a need for urgent surgery after PCI. As such, this indicates a complex clinical scenario, and the outcomes of these patients may be influenced by factors beyond the initial revascularization strategy. Therefore, the variable CABG was not selected for the analysis to allow for a more focused analysis of the impact of the revascularization strategy on outcomes specific to the initial MI presentation. Accordingly, we used the terms ‘PCI’ and ‘revascularization’ interchangeably. This clarity may be essential for readers to correctly interpret our findings. Smoking habits were self-reported. Hypertension, hypercholesterolaemia, and diabetes were assessed by documentation of medical history prior to admission in the database (Supplementary material online).

### Statistical analysis

Baseline characteristics were reported as percentages for categorical variables and means with standard deviation for continuous variables (*Table 1*). Comparisons between groups were made either by the Pearson chi-square test for baseline categorical variables or the Student's *t*-test for continuous variables, as appropriate. We had complete data on sex, index events, and outcomes. Some patients had missing data on other variables (Supplementary material online, *Table S1*). We used Multiple Imputation with Chained Equation as the imputation method to treat missing data (Supplementary material online).^8^ Estimates of the adjusted risk ratios (RRs) and associated 95% CIs were obtained using inverse probability weighting models. Inverse probability weights were calculated using the propensity score to create a sample in which the distribution of measured baseline covariates was independent from sex (Supplementary material online).^9^ Standardized differences (SD) after weighting were calculated to ensure balanced treatment groups with respect to baseline characteristics. Groups were considered balanced when the SD was less than 10% (Supplementary material online).^10^ Comparisons of outcomes between groups were made by two-sided *P-*value of <0.05. The characteristics incorporated into the models for the outcome of 30-day mortality are shown in Supplementary material online, *Table S2*. Variables included demographics (age and sex), cardiovascular risk factors (diabetes, hypertension, hypercholesterolaemia, current smokers, former smokers), medical history (prior angina pectoris, prior MI, prior PCI, prior CABG, peripheral artery disease), and clinical features on hospital presentation (systolic blood pressure, heart rate, serum creatinine levels). Sensitivity analyses were conducted to estimate the effect of revascularization in the overall population with MI and in the two subsets of patients presenting to the hospital with different baseline ischaemic risk: STEMI and NSTEMI. Moreover, to assess significant heterogeneity of outcomes in function of sex and delay from symptom onset to hospital presentation, we made statistical comparisons across 2 delay cohorts (<120 and ≥120 min). To account for variations in propensity scores due to the impact of centres and locations in different countries, we include the random intercept effects on the propensity models using the approach described by Abadie *et al*.^11^ We reported the coefficient estimates, clustered adjusted standard errors, T statistics, and corresponding *P*-values in the Supplementary material online, *Tables S3–S5*. Finally, to minimize concern about the comparison of outcomes in subgroups, estimates were compared by a test of interaction on the log scale.^12^ A *P*-value <0.05 was taken to indicate that the difference between the effects in women and men was unlikely to have occurred simply by chance (Supplementary material online). Our primary focus was on understanding the differences between two groups (MICs and in HICs) in relation to sex. Given our research focus, we choose not to conduct post-hoc multiple analyses to understand the relationships between different countries within the ‘MIC’ and ‘HIC’ groups. We considered ‘HICs’ as one category and ‘MICs’ as the other category to make the binary variable. All statistical analyses were performed using R, version 3.4.4.

**Table 1:** Baseline characteristics of the overall population stratified by sex and economic status

	Women (*n* = 6669)			Men (*n* = 15 418)		
Characteristics	Middle income countries (*n* = 4192)	High income countries (*n* = 2477)	Standardized difference	Middle income countries (*n* = 9429)	High income countries (*n* = 5989)	Standardized difference
Age, mean ± SD, years	65.8 ± 11.5	68.0 ± 12.0	−0.19^a^	60.3 ± 11.5	63.5 ± 12.3	−0.27^a^
**Cardiovascular risk factors**						
Diabetes, *n* (%)	1332 (31.8)	621 (25.1)	0.15^a^	2120 (22.5)	1241 (20.7)	0.04^a^
Hypertension, *n* (%)	3252 (77.6)	1668 (67.3)	0.23^a^	6151 (65.2)	3461 (57.8)	0.15^a^
Hypercholesterolemia, *n* (%)	1793 (42.8)	1020 (41.2)	0.03	3906 (41.4)	2403 (40.1)	0.03
Current smokers, *n* (%)	1302 (31.1)	672 (27.1)	0.09^a^	4454 (47.2)	2337 (39.0)	0.17^a^
Former smokers, *n* (%)	115 (2.7)	428 (17.3)	−0.50^a^	604 (6.4)	1610 (26.9)	−0.57^a^
**Clinical history of CHD**						
Prior angina pectoris, *n* (%)	792 (18.9)	322 (13.0)	0.16^a^	1357 (14.4)	883 (14.7)	−0.01
Prior myocardial infarction, *n* (%)	567 (13.5)	365 (14.7)	−0.03	1414 (15.0)	943 (15.7)	−0.02
Prior PCI, *n* (%)	463 (11.0)	180 (7.3)	0.13^a^	1149 (12.2)	525 (8.8)	0.11^a^
Prior CABG, *n* (%)	54 (1.3)	82 (3.3)	−0.13^a^	214 (2.3)	229 (3.8)	−0.09^a^
**Clinical history of CVD**						
Peripheral artery disease, *n* (%)	60 (1.4)	98 (4.0)	−0.16^a^	137 (1.5)	267 (4.5)	−0.18^a^
**Medications before index event**, *n* (%)	2858 (68.2)	1466 (59.2)	0.19^a^	5289 (56.1)	3005 (50.2)	0.12^a^
Aspirin, *n* (%)	1396 (33.3)	712 (28.7)	0.10^a^	2632 (27.9)	1592 (26.6)	0.03
P2Y_12_ inhibitors, *n* (%)	351 (8.4)	267 (10.8)	−0.08^a^	741 (7.9)	674 (11.3)	−0.11^a^
ACE inhibitors/ARBs, *n* (%)	2322 (55.4)	990 (40.0)	0.31^a^	4042 (42.9)	1924 (32.1)	0.22^a^
β blockers, *n* (%)	1603 (38.2)	761 (30.7)	0.16^a^	2837 (30.1)	1554 (25.9)	0.09^a^
Statins, *n* (%)	884 (21.1)	683 (27.6)	−0.15^a^	1889 (20.0)	1562 (26.1)	−0.14^a^
**Clinical presentation**						
STEMI, *n* (%)	2885 (68.8)	1428 (57.7)	0.23^a^	6690 (71.0)	3465 (57.9)	0.27^a^
SBP at admission, mean ± SD, mmHg	139.3 ± 28.6	138.2 ± 29.2	0.04	139.9 ± 27.1	138.7 ± 32.8	0.04^a^
HR at admission, mean ± SD, bpm	82.1 ± 19.5	80.3 ± 21.7	0.08^a^	81.5 ± 18.8	79.0 ± 20.0	0.12^a^
Serum creatinine at admission, mean ± SD, mg/dL	1.0 ± 0.5	1.0 ± 0.6	−0.08^a^	1.1 ± 0.7	1.1 ± 0.7	−0.04^a^
**Revascularization procedures**, *n* (%)	3161 (75.4)	1538 (62.1)	0.29^a^	7844 (83.2)	4077 (68.1)	0.36^a^
Fibrinolysis, *n* (%)	208 (5.0)	191 (7.7)	−0.11^a^	615 (6.5)	611 (10.2)	−0.13^a^
PCI, *n* (%)	3024 (72.1)	1388 (56.0)	0.34^a^	7540 (80.0)	3667 (61.2)	0.42^a^
**Medications on admission**						
Aspirin, *n* (%)	4056 (96.8)	2374 (95.8)	0.05	9248 (98.1)	5798 (96.8)	0.08^a^
P2Y_12_ inhibitors, *n* (%)	3694 (88.1)	1689 (68.2)	0.49^a^	8253 (87.5)	4354 (72.7)	0.38^a^
**Medications during hospital stay**						
ACE inhibitors/ARBs, *n* (%)	3159 (75.4)	1765 (71.3)	0.09^a^	7159 (75.9)	4442 (74.2)	0.04^a^
β blockers, *n* (%)	3100 (74.0)	1808 (73.0)	0.02	7025 (74.5)	4506 (75.2)	−0.01
Statins, *n* (%)	3896 (92.9)	2237 (90.3)	0.09^a^	9011 (95.6)	5528 (92.3)	0.13^a^
**Outcomes**						
30-day mortality, *n* (%)	412 (9.8)	174 (7.0)	0.10^a^	512 (5.4)	234 (3.9)	0.07^a^

Values are mean ± standard deviation (SD) or numbers (percentages), unless otherwise specified.

ACE, angiotensin converting enzyme; ARBs, angiotensin receptor blockers; bpm, beats per minute; CABG, coronary artery bypass graft; CHD, coronary heart disease; CVD, cardiovascular disease; HR, heart rate; PCI, percutaneous coronary intervention; SBP, systolic blood pressure; STEMI, ST-segment elevation myocardial infarction.

^a^Denotes *P*-values <0.05.

## Results

### European cardiovascular disease statistics data

Data of the European Cardiovascular Disease Statistics by sex and country are summarized in *Table 2*. There was heterogeneity among countries within income categories. The relation between prevalence and death rates of IHD for 100 000 inhabitants varied widely both in men and women from MICs (4.6% and 4.7%, respectively, in Montenegro vs. 24.7% and 37.5%, respectively, in the Republic of Moldova) and in men and women from HICs (8.7% and 7.8%, respectively in the UK vs. 9.9% and 12.8%, respectively, in Croatia). There was not a clear trend in outcome rates by sex and income levels. The women-to-men rate ratios ranged from 0.90 in the UK to 1.52 in Moldova. The mean value for HICs was 1.20 and for MICs 1.38. The overall mean value was 1.27.

**Table 2:** Age-standardized rate ratios (women to men) for IHD per 100 000 inhabitants

	Men			**Women**			
Country (income group)	Age-standardized IHD mortality rate	Age-standardized IHD prevalence rate	**%**	**Age-standardized IHD mortality rate**	**Age-standardized IHD prevalence rate**	**%**	**Rate ratio** **Women to men**
**Middle-income countries**							
Bosnia and Herzegovina	185	3267	5.7	132	1741	7.6	1.33
North Macedonia	188	3111	6.0	103	1560	6.6	1.09
Republic of Moldova	898	3639	24.7	717	1913	37.5	1.51
Montenegro	138	2967	4.6	72	1518	4.7	1.01
Serbia	194	3589	5.4	125	1861	6.7	1.24
Overall middle-income countries	1603	16 573	9.7	1149	8593	13.4	1.38
**High-income countries**							
Croatia	341	3458	9.9	244	1905	12.8	1.29
Hungary	479	3847	12.4	315	1971	15.9	1.28
Italy	148	1507	9.8	83	824	10.1	1.02
Lithuania	700	4394	15.9	429	2263	18.9	1.18
Romania	364	3778	9.6	250	2041	12.2	1.27
United Kingdom	177	2037	8.7	87	1117	7.8	0.89
Overall high-income countries	2209	19 021	11.6	1408	10 121	13.9	1.19
*Overall (MIC and HIC)*	3812	35 594	10.7	2557	18 714	13.7	1.27

To calculate the rate ratio, first we calculated the death rate per 100 000 inhabitants among the prevalence rate per 100 000 inhabitants for each country (here expressed as percentages). Next, the rate for the group of primary interest (women) was divided by the rate for the comparison group (men). A rate ratio of 1.0 indicates equal rates in the two groups, a rate ratio greater than 1.0 indicates an increased risk for women, and a rate ratio less than 1.0 indicates a decreased risk for women.

Source of data: European Cardiovascular Disease Statistics.^7^

IHD, ischaemic heart disease; HIC, high income country; MIC, middle income country.

### ISACS archives registry data

A total of 22 087 MI patients met inclusion criteria. There were 6669 (30.2%) women. We identified 14 468 patients with STEMI (29.8% women) and 7619 patients with NSTEMI (30.9% women) (*Figure 1*). Women with STEMI were less likely to have a timely hospital presentation (<120 min) than men, both in MICs and HICs (Supplementary material online, *Figure S1*). Nonetheless, we found that a significantly greater proportion of women and men from MICs had earlier presentations (<120 min) compared with those from HICs (25.3% vs. 19.3% and 31.1% vs. 23.3%, respectively).

### Baseline clinical characteristics

We assessed the baseline characteristics of the patient cohort within the two groups of countries categorized by income level (*Table 1*). Women and men from MICs were significantly (SD > 10%) younger than those from HICs. The baseline cardiovascular risk was higher in patients from MICs compared with those from HICs. Rates of hypertension were higher in MICs (SD > 10%) compared to HICs for both women (77.6% vs. 67.3%) and men (65.2% vs. 57.8%). A larger proportion of women and men from MIC reported a history of prior PCI (11.0% and 12.2%, respectively) as compared with the corresponding proportions of patients from HIC (7.3% and 8.8%, respectively). Patients from MIC were more likely to present with STEMI compared with those from HIC (68.8% vs. 57.7%, SD = 0.23 in women and 71.0% vs. 57.9%, SD = 0.27 in men) and received more revascularization procedures by PCI (72.1% vs. 56.0%, SD = 0.34 in women and 80.0% vs. 61.2%, SD = 0.42 in men).

We also observed substantial sex-specific differences between countries at different country-income levels. Higher rates of diabetes were more common among women from MICs than those from HICs (31.8% vs. 25.1%, SD = 0.15). By contrast, we found no significant differences between country income levels and rates of diabetes in men (22.5% vs. 20.7%, SD = 0.04). Regarding medications before the index event, there was a consistent pattern of greater use of ACE-inhibitors/ARBs and lower use of statins in women and men from MICs compared with those from HICs. Use of β-blockers before index events was greater in women from MIC compared with those from HIC (38.2% vs. 30.7%, SD = 0.16). Use of β-blockers in men did not vary significantly by country-income level (30.1% vs. 25.9%, SD = 0.09). Overall, there was an imbalance of clinical covariates between women and men, as shown by the optimized regression coefficient β and constant term (α) for the logistic regression (Supplementary material online, *Tables S3–S5*).

### Outcomes among patients with myocardial infarction

**Figure 2:**
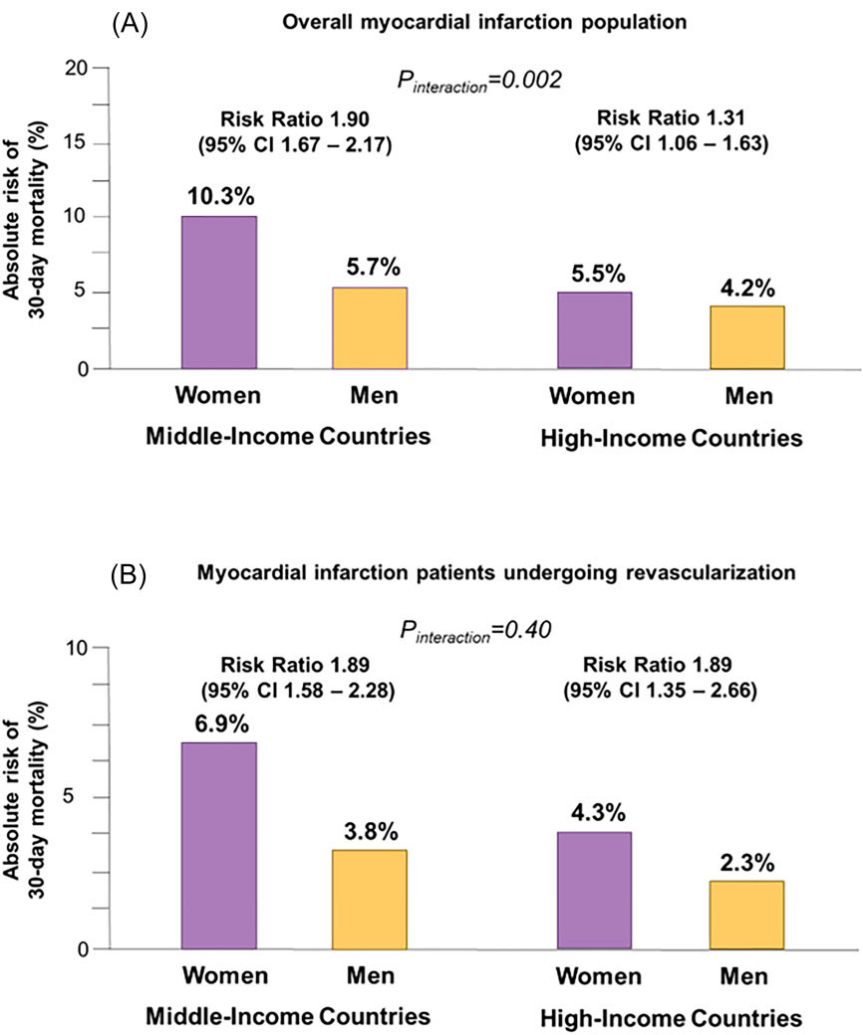
Absolute and relative risk of 30-day mortality stratified by sex and income level in the overall population (panel A) and in patients undergoing revascularization (panel B).

We examined the association between sex and outcomes of MI using a parametric balancing strategy by weighting. Covariates were well balanced among women and men (Supplementary material online, *Table S2*). Women were at increased risk of the primary outcome of death compared with men (*Figure 2*). Significant variations in outcomes were observed between the two populations categorized by different country-income levels. Sex differences in death were smaller in HIC [absolute difference 1.3%; RR, 1.31 (95% CI, 1.06–1.63)] compared with MIC [absolute difference 4.6%; RR, 1.90 (95% CI, 1.67–2.17), *P*_interaction _= 0.002] (Supplementary material online, *Tables S2* and *S6*).

### Outcomes among patients with STEMI and NSTEMI

**Figure 3:**
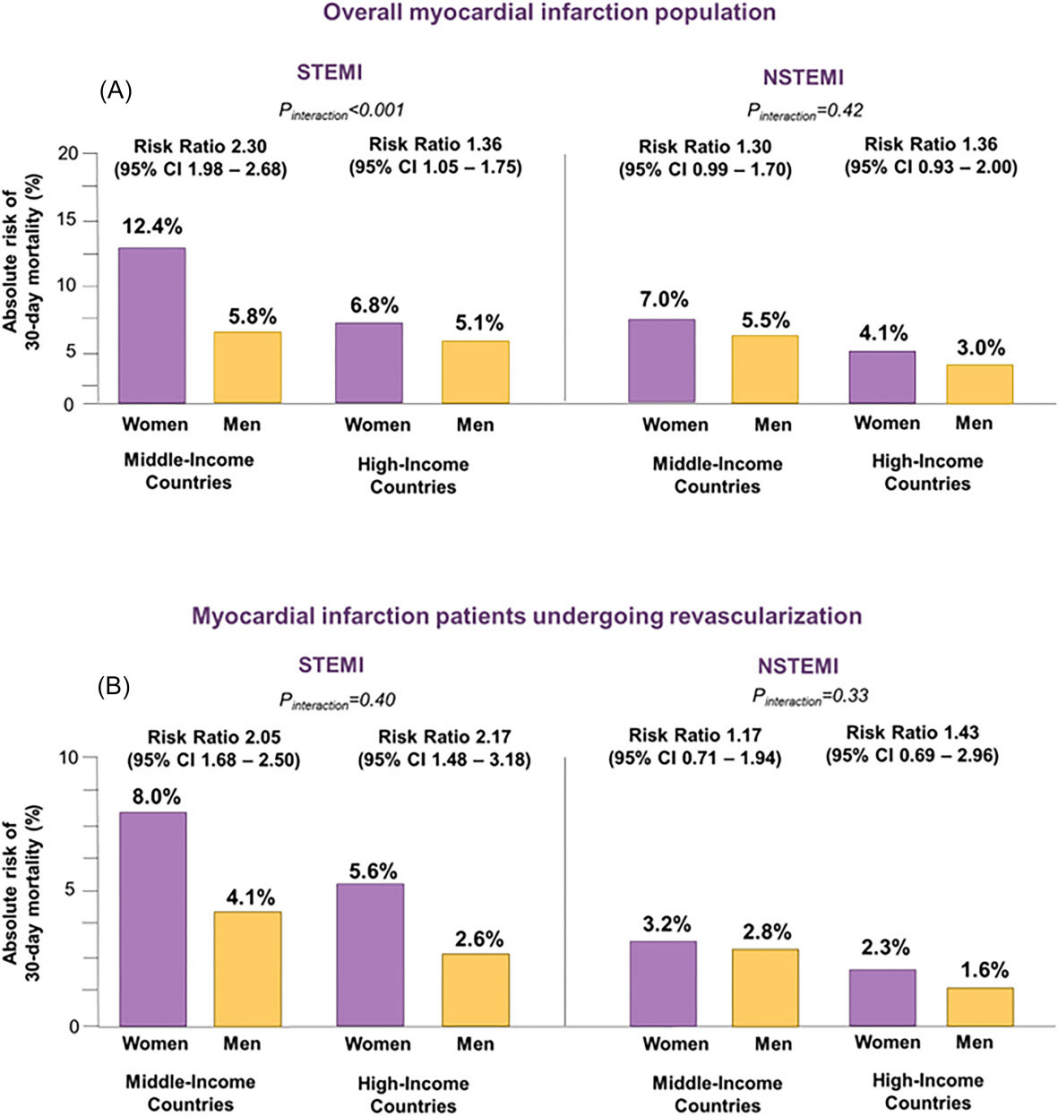
Thirty-day mortality in MI patients stratified by sex and national income level group. Absolute and relative risk of 30-day mortality stratified by sex and income level in the overall population of STEMI and NSTEMI (panel A) and in patients undergoing revascularization (panel B). NSTEMI, non-ST-segment elevation myocardial infarction; STEMI, ST-segment elevation myocardial infarction.

A different perspective of risk was provided by stratifying patients into the two categories of clinical presentation: STEMI and NSTEMI (*Figure 3A*). After STEMI, the proportion of women who died within 30 days was around twice more than men in MICs [12.4% vs. 5.8%; RR, 2.30 (95% CI, 1.98–2.68)]. The difference was much lower in HICs [6.8% vs. 5.1%; RR, 1.36 (95% CI, 1.05–1.75)]. The income differences in women-to-men RRs were significantly different (*P*_interaction_ <0.001) (Supplementary material online, *Tables S7* and *S8*). By contrast, outcomes for NSTEMI were relatively uniform among women and men and between the two groups of countries. RR: 1.30; 95% CI: 0.99–1.70 for MIC and RR, 1.36 (95% CI, 0.93–2.00) for HIC; *P*_interaction_ = 0.43 (Supplementary material online, *Tables S9* and *S10*).

### Outcomes after revascularization for myocardial infarction

As sex disparities in the utilization of coronary revascularization procedures have been a topic of concern in prior studies, we restricted the population of patients to those who received revascularization therapy. *Figure 2* displays the results of estimates that examined the association between sex and 30-day mortality in the overall population of MI undergoing revascularization. Adjusted for the observed patient factors, revascularization was consistently associated with lower 30-day mortality as compared with that of the overall population (*Figure 2*A). Still, the rate of death in patients who underwent revascularization remained higher in women than men in both MICs [6.9% vs. 3.8%; RR, 1.89 (95%CI, 1.58–2.28)] and HICs [4.3% vs. 2.3%; RR, 1.89 (95%CI, 1.35–2.66), *P*_interaction _= 0.40] (*Figure 2*B and Supplementary material online, *Table S11*).

### Comparison of outcomes with and without revascularization

As we compared the 30-day mortality of patients with revascularization (*Figure 2*B) with the data reported in *Figure 2*A, which is a mix of patients who had PCI and those who did not, we performed a further analysis by comparing the rates of mortality in those with and without revascularization. Overall, the adjusted mortality rates in women from MICs were 8.7% vs. 12.7% in those who did and did not undergo revascularization [RR, 0.66 (95% CI, 0.53–0.82)]. The corresponding rates for HICs were 5.5% and 8.6% [RR, 0.61 (95% CI, 0.45–0.84), *P*_interaction _= 0.34] (Supplementary material online, *Tables S12* and *S13*). Similar patterns were observed in men [in MICs: from 7.8% to 4.3%; RR, 0.53 (95% CI, 0.43–0.64); in HICs: from 5.1% to 2.6%; RR, 0.50 (95% CI, 0.38–0.66), *P*_interaction _= 0.36] (Supplementary material online, *Tables S14* and *S15*).

### Outcomes after revascularization for STEMI and NSTEMI

Subgroup analyses of patients undergoing revascularization by type of MI revealed consistent sex differences in mortality for STEMI but not for NSTEMI (*Figure 3*B). Sex differences in mortality persisted for patients with STEMI in both MICs [8.0% vs. 4.1%; RR, 1.17 (95%CI, 0.71–1.94)] and HICs [5.6% vs. 2.6%; RR, 1.43 (95%CI: 0.69–2.96); *P*_interaction _= 0.40] (Supplementary material online, *Tables S16* and *S17*). By contrast, there was no sex difference in mortality in patients with NSTEMI [RR, 1.17 (95%CI, 0.71–1.94) in MICs and RR, 1.43 (95%CI, 0.69–2.96) in HICs; *P*_interaction _= 0.33] (Supplementary material online, *Tables S18* and *S19*).

## Discussion

In this propensity score-based analysis of patients experiencing MI in countries with different socio-economic profiles, we found some clinically relevant findings. First, we observed that women in MICs have nearly a two-fold higher 30-day mortality following MI compared with men, even after extensive adjustment for confounding variables. This difference was much lower in HICs. Second, we showed that the breakdown of data into different types of MI reveals a complex pattern of risk. Women with STEMI still have a higher rate of mortality compared with men, with a greater sex gap in MICs. On the opposite, outcomes for NSTEMI are relatively uniform among women and men and between the two groups of countries. Third, we found that, even after timely revascularization, there was a persistence of much higher absolute mortality rates in women from MICs compared with those from HICs, suggesting that, beyond revascularization, there may be other underlying factors contributing to a higher baseline risk of death for women in MICs. Fourth, we identified diabetes as an effect modifier for the effects of revascularization. Its prevalence in women from MICs may lead to higher absolute mortality rates compared with women from HICs. These findings, therefore, have implications not only for individual patient care in MICs but also for global health strategies.

There are large sex variations in regional mortality for IHD in Europe, as reported by the European Cardiovascular Disease Statistics,^7^ and this is a matter of concern. Age-standardized mortality in women who have an established diagnosis of IHD is higher than that for men in almost all of the European countries surveyed in this study, with a mean rate ratio of approximately 1.2 (*Table 2*). In general, the higher the country’s income level, the lower the sex difference in cardiovascular outcomes. Yet, there are large disparities even across countries with similar incomes.^13^ The finding emphasizes the need for understanding which specific factors are contributing to these variations. Whether differences among women and men reflect differences in patient characteristics, inequalities of care or, to some extent, patient selection is a matter of debate. The European Cardiovascular Disease Statistics is limited by the fact that estimates are derived through combining data from a variety of sources, including survey data, outpatient and inpatient visits of angina, and first-acute MI. The reliability of some of the above-reported estimates can be improved only by large studies involving multiple European countries at different economic levels and patient-level data.

The ISACS Archives registry focused on outcomes after MI. Consistent with European Cardiovascular Disease Statistics results,^7^ the data of the current investigation showed that the sex differences in MI outcomes vary considerably between MICs and HICs, even after adjustment for confounding factors. (*Figure 2*A). Notably, the outcome differences were largely driven by differences in the rates of mortality of female sex. As illustrated in *Figure 2*A, the absolute percentage of deaths among women in MICs remarkably exceeded that of women in HICs (10.3% vs. 5.5%), whereas differences among men in MICs and HICs were much smaller (5.5% vs. 4.2%). There are, therefore, specific factors affecting women's outcomes in MI that vary across different economic settings, which may include access to healthcare facilities, delayed presentation, and differences in baseline characteristics.

Although we cannot identify the precise mechanism of the association between female sex and worse outcomes in MICs, our data suggest that some hypotheses can be discounted. It is commonly believed that limited resources and healthcare infrastructure in some MICs could impact access to revascularization procedures, particularly in women. However, in contrast with this thinking, we found that the number of revascularization procedures for MI was higher in patients from MICs compared with those from HICs in both women (72.1% vs. 56.0%) and men (80.0% vs. 61.2%; *Table 1*). The long-tErm follow-uP of antithrombotic management patterns In acute CORonary syndrome patients (EPICOR) and EPICOR Asia registries analysed results across eight pre-specified regions, including various parts of Europe, Latin America, and Asia. Their findings show primary PCI rates closely aligned with ours, ranging from approximately 65% in Eastern Europe to 64.5% in Southern Europe and 65.6% in Northern Europe. Additionally, the median time to PPCI within the first 12 h from symptom onset was comparable across these regions, supporting our observations.^14,15^ The association does not reflect a lower likelihood of women from MICs to receive guideline-recommended treatments,^16^ since women from MICs received on average more evidence-based medications than those from HICs. Studies have also suggested that women more frequently present with atypical symptoms than do men, which might contribute to delays in subsequent care.^17^ Although our data confirm different delays between women and men (Supplementary material online, *Figure S1*), we also found that a significantly lower proportion of women from MICs had delayed presentation compared with those from HICs. As such, delayed presentation cannot be the reason of the observed disparities in outcomes of women between MICs and HICs.

A possible mechanism for the increased risk of death after MI among women in MICs compared with HICs may involve the varying baseline ischaemic risk of patients. The most specific indicator of this higher baseline risk was the likelihood of STEMI, which was consistently higher among patients from MICs compared with those of HICs. Baseline risk differences for STEMI were observed both in women (68.8% vs. 57.7%; *Table 1*) and men (71.0% vs. 57.9%), and this needs to be appreciated. Data from the National Heart, Lung, and Blood Institute Dynamic Registry found that STEMI is associated with a higher likelihood of early death than NSTEMI.^18^ It is also well-documented that there are sex differences in the outcomes following STEMI, with early mortality rates being higher in women compared with men, while NSTE-ACS/NSTEMI outcomes are comparable or worse in men.^19–21^ It follows that the disadvantage of MI was two‐fold for women in MICs: a greater percentage of women had STEMI, and women with STEMI had higher mortality than men with STEMI.

Still, we remain with an unsolved issue. Why women with STEMI in MICs have a higher rate of mortality compared with women with STEMI in HICs? As previously shown, instances of limited access to revascularization procedures and delayed presentation were not solely attributable to the country’s income level, and, as such, they cannot explain this disparity. Unravelling the complexities of this disparity therefore required a nuanced examination of various potential contributing factors, including the quality of care and differences in the prevalence of cardiovascular risk factors.

The observation that, following revascularization, women and men experienced approximately equal proportionate reductions in mortality indicates that treatment was similarly effective across MICs and HICs (*Figure 3*). As such, the persistence of a much higher absolute mortality rate in revascularized women from MICs compared with those from HICs (8.0% vs. 5.6%) points to a higher baseline risk of death in women in lower-income countries. The focus of disparity, therefore, shifted from treatment to risk factors that may contribute to differences in baseline risk and outcomes.

In the current study, we observed significantly greater rates of diabetes among women from MICs compared with those from HICs (31.8% vs. 25.1%) (*Table 1*). By contrast, the prevalence among men remained relatively constant across the two groups of countries (22.5% vs. 20.7%). Diabetes is a known risk factor for adverse cardiovascular events,^22,23^ and its higher prevalence in women from MICs may contribute to poorer outcomes. Accordingly, prior work has shown that the hazard of early mortality after STEMI is much more remarkable in women with diabetes than in women without diabetes or men with diabetes.^23,24^ Dietary patterns differ in regions with various income levels, and the context in which foods are accessed differs markedly. Perhaps consumption of an unhealthy diet and other behavioural risk factors in MICs could be associated with the observed increased risk of diabetes in women.^13^

Prior epidemiological studies examining cardiovascular risk and mortality in MICs have predominantly focused on regions outside of Europe, including South America, Asia, and Africa.^25,26^ The EPICOR and EPICOR Asia registries, encompassing 32 countries, featured only a single MIC from Europe, contrasting with 13 European HICs.^27^ Furthermore, a recent serial cross-sectional cohort study by the International Health Systems Research Collaborative spanning six countries included only two from Europe—both classified as HICs (England and the Netherlands), making direct comparisons between European MICs and HICs challenging.

## Limitations

The study findings should be interpreted with the following limitations in mind. First, as an observational study, we cannot completely exclude residual unmeasured confounding, such as quality of care, socioeconomic and environmental variables. Concern about bias in baseline characteristics and interventional strategies was minimized using a parametric balancing strategy by inverse probability weighting. Second, the sites that contributed to this analysis are mainly from urban areas.^3,4^ Therefore, caution is needed in interpreting our data as being representative of each country, even though the rates of death for MI in the European MICs versus HICs correlated closely with the national rates of death reported by the European Cardiovascular Disease Statistics.^7^ Our approach to socioeconomic status was constrained by the availability and granularity of data, leading us to rely on the World Bank's country-based income classifications. This method can mask important income differentials at the patient level within any country. Disparities may result from various factors, including differences in employment opportunities, education, geographic location, social class, and other social determinants of cardiovascular health. Consequently, relying solely on a country's income classification can overlook the socioeconomic variations that exist within that country.^28^ Fourth, we cannot rule out that a large number of women and men may have died before presentation to hospitals, especially in the MIC countries. As well, some of the risk factors were ascertained by the general practitioner, which might have led to errors in the dataset. Nonetheless, this was the closest attainable estimate of risk factors, as blood pressure and glycaemic values are potentially confounded by the concurrence of MI. Finally, STEMI represented the majority of MI admitted to intensive cardiac care units/cardiology departments at the participating European centres, which usually is not seen in real-world practice of United States, where NSTEMI predominates.^29^ Yet, the observed rates of STEMI and NSTEMI in our study are in keeping with those reported by the Euro Heart Survey^30^ and the French registry of Acute ST elevation or non-ST-elevation Myocardial Infarction (FAST-MI) 2010.^31^ This finding highlights a notable difference in the MI presentation patterns between the two regions.

## Conclusions

The observation of seemingly greater access to timely and suitable revascularization procedures in MICs, where the disparity in mortality between sexes from STEMI is more pronounced, contradicts the assumption that greater economic resources would invariably result in improved healthcare outcomes. This paradox suggests that disparities in mortality among women and men are not solely explained in the context of sex inequity in revascularization and that additional factors contribute to excess mortality. Countries with a low economic level have a greater burden of risk factors than those with a high economic level. Recognizing the role of cardiovascular risk factors in contributing to sex disparities in baseline risk after STEMI highlights the importance of strengthening not only the healthcare system for acute cardiovascular events but also preventive healthcare initiatives for managing the underlying risk factors that contribute to adverse outcomes. In our exploratory analysis, the presence of diabetes could potentially influence the observed differences in outcomes between men and women following STEMI. Implementing primary prevention programs is, therefore, warranted in MICs and may result in a viable opportunity to reduce the sex gap in mortality.

The lower rates of death in both men and women undergoing revascularization may be indicative of the effectiveness of these interventions in reducing mortality associated with STEMI. These results, therefore, caution against an oversimplified interpretation of the absence of association between revascularization and reduced mortality in women with MI, as the relation between revascularization and sex-specific outcome needs to be tailored to the type of MI.

## Supplementary Material

qcae035_Supplemental_File

## Data Availability

To guarantee the confidentiality of personal and health information, only the authors have had access to the data during the study. The source codes for this manuscript are uploaded on GitHub.
