# Supplementary material for: Coronary revascularization and sex differences in cardiovascular mortality after myocardial infarction in 12 high and middle-income European countries
Source: Eur Heart J Qual Care Clin Outcomes. 2024 May 7;11(6):719–29. doi: 10.1093/ehjqcco/qcae035 (PMC12445642; doi:10.1093/ehjqcco/qcae035)
Supplement: qcae035_Supplemental_File [file qcae035_supplemental_file.docx]

**Supplementary material**

**Coronary revascularization and sex differences in cardiovascular mortality after myocardial infarction in 12 high and middle-income European countries**

**Table of contents**

[**SUPPLEMENTAL METHODS** 3](#_Toc159866618)

[**Centers and Countries of the ISACS-TC and EMMACE-3X registries** 3](#_Toc159866619)

[**Risk factors definitions** 6](#_Toc159866620)

[**Multiple Imputation using Chained Equation (MICE) algorithm** 7](#_Toc159866621)

[**Inverse Propensity Score Weighting Analysis** 7](#_Toc159866622)

[**Computation of Relative Risk and its Confidence Interval** 8](#_Toc159866623)

[**Comparison of means and prevalences in the weighted sample** 9](#_Toc159866624)

[**Interaction test** 10](#_Toc159866625)

[**SUPPLEMENTAL RESULTS** 11](#_Toc159866626)

[**Interaction tests** 11](#_Toc159866627)

[**Figure S1.** Rates of STEMI patients presenting <2 hours from symptoms onset by sex and income categories 12](#_Toc159866628)

[**Table S1.** Rate of missing values for covariates used for inverse probability weighting models 13](#_Toc159866629)

[**Table S2.** Inverse probability weighting: clinical factors and outcomes stratified by sex and economic status in patients with myocardial infarction 14](#_Toc159866630)

[**Table S3.** General logistic regression and regression coefficients in the propensity score model in the overall study population (women versus men) 16](#_Toc159866631)

[**Table S4.** General logistic regression and regression coefficients in the propensity score model in women (high income countries vs middle income countries) 17](#_Toc159866632)

[**Table S5.** General logistic regression and regression coefficients in the propensity score model in men (high income countries vs middle income countries) 18](#_Toc159866633)

[**Table S6.** Interaction test: calculations for comparing two estimated risk ratios (women vs men) for 30-day mortality by inverse probability weighting: high-income versus middle-income countries 19](#_Toc159866634)

[**Table S7.** Inverse probability weighting: clinical factors and outcomes stratified by sex and economic status income in STEMI patients 20](#_Toc159866635)

[**Table S8.** Interaction test: calculations for comparing two estimated risk ratios (women vs men) for 30-day mortality by inverse probability weighting: high-income countries versus middle-income countries in STEMI patients 22](#_Toc159866636)

[**Table S9.** Inverse probability weighting: clinical factors and outcomes stratified by sex and economic status in NSTEMI patients 23](#_Toc159866637)

[**Table S10.** Interaction test: calculations for comparing two estimated risk ratios (women vs men) for 30-day mortality by inverse probability weighting: high-income countries versus middle-income countries in NSTEMI patients 25](#_Toc159866638)

[**Table S11.** Inverse probability weighting: clinical factors and outcomes stratified by sex and economic status in patients with myocardial infarction undergoing revascularization 26](#_Toc159866639)

[**Table S12.** Inverse probability weighting: clinical factors and outcomes stratified by economic status and revascularization choice in women with myocardial infarction. 28](#_Toc159866640)

[**Table S13.** Interaction test: calculations for comparing two estimated risk ratios (No PCI versus PCI) for 30-day mortality by inverse probability weighting: Women 30](#_Toc159866641)

[**Table S14.** Inverse probability weighting: clinical factors and outcomes stratified by economic status and revascularization choice in men with myocardial infarction 31](#_Toc159866642)

[**Table S15.** Interaction test: calculations for comparing two estimated risk ratios (No PCI versus PCI) for 30-day mortality by inverse probability weighting: Men 33](#_Toc159866643)

[**Table S16.** Inverse probability weighting: clinical factors and outcomes stratified by sex and economic status in STEMI patients undergoing revascularization 34](#_Toc159866644)

[**Table S17.** Interaction test: calculations for comparing two estimated risk ratios (women vs men) for 30-day mortality by inverse probability weighting: high-income countries versus middle-income countries in STEMI patients undergoing revascularization 36](#_Toc159866645)

[**Table S18.** Inverse probability weighting: clinical factors and outcomes stratified by sex and economic status in NSTEMI patients undergoing revascularization 37](#_Toc159866646)

[**Table S19.** Interaction test: calculations for comparing two estimated risk ratios (women vs men) for 30-day mortality by inverse probability weighting: high-income countries versus middle-income countries in NSTEMI patients undergoing revascularization 39](#_Toc159866647)

[**REFERENCES** 40](#_Toc159866648)

# **SUPPLEMENTAL METHODS**

**The International Survey of Acute Coronary Syndromes (ISACS) Archives.**

The ISACS Archives network (NCT04008173) is part of ISACS TC (NCT01218776) health care program. It is a collaborative network of research centers that support rapid development of new scientific information and analytic tools. The ISACS Archives uses an established informatics infrastructure, hosted and managed by the ISACS TC registry (NCT01218776)^1^ and the Department of Electrical and Computer Engineering, University of California, Los Angeles, which enables sharing of data. The ISACS Archives includes sites in which investigators are committed to collecting good-quality data without a strict proportionate sampling. Registries enrolled in the ISACS Archives use data definition for the measures/experiments that are harmonized to the standard variables of the ISACS –TC.^1^ Participation in the research network does not eliminate the ability of any individual patient registry from analyzing only the data from the registry alone.

**Centers and Countries of the ISACS-TC and EMMACE-3X registries**

As the aim of the current investigation was to analyze the relation between cardiovascular outcomes and country-level income from 2010 to 2021, we identified two large clinical registries providing such information, namely the ISACS-TC (NCT01218776)^1^ and the EMMACE-3X (Long-term Follow-up of Health-Related Quality of Life in Patients with Acute Coronary Syndrome; NCT01955525).^2^ In brief, the ISACS-TC registry collected data from 40 centers in 12 European countries: Bosnia and Herzegovina, Croatia, Italy, Kosovo, Lithuania, Hungary, North Macedonia, Moldova, Montenegro, Romania, Serbia, and United Kingdom. Among these sites, there are 22 tertiary health care services providing percutaneous coronary intervention (PCI). The EMMACE-3X gathered routine clinical information from 47 hospitals in England. Cardiovascular facilities including PCI were available in 33 hospitals.

| **Registry** | **Country** | **Center** |
| --- | --- | --- |
| **ISACS-TC** |  |  |
|  | **Bosnia and Herzegovina** |  |
|  |  | Clinical Center of Banja Luka* |
|  |  | Clinical Center University of Sarajevo* |
|  |  | Opšta Bolnica:"Sveti Vracevi" |
|  |  | Opšta Bolnica, Gradiška |
|  |  | Klinički Centar, Kasindo |
|  |  | Dom Zdravlja “Dr Mladen Stojanovć”, Bačka Palanka |
|  |  | Opšta Bolnica “Sveti Apostol Luka”, Doboj |
|  |  | Univesity Clinical Hospital Mostar* |
|  | **Croatia** |  |
|  |  | University Hospital Centre of Zagreb, Zagreb* |
|  |  | Clinical Hospital Merkur, Zagreb* |
|  |  | Clinical Hospital Dubrava, Zagreb |
|  | **Italy** |  |
|  |  | Sapienza-Cuore Grossi Vasi, Rome |
|  | **Kosovo** |  |
|  |  | Clinical Center of Kosovo, Prishtina |
|  | **Lithuania** |  |
|  |  | Hospital of Lithuanian University of Health Sciences, Kaunas* |
|  | **Macedonia** |  |
|  |  | University Clinic of Cardiology, Skopje* |
|  | **Hungary** |  |
|  |  | University of Pecs, Medical School, 1st Department of Medicine, Division of Cardiology |
|  | **Moldova** |  |
|  |  | Hospital Center of Cardiology; Institute of Cardiology, Chișinău |
|  | **Montenegro** |  |
|  |  | Clinical Center of Montenegro, Podgorica) |
|  |  | General Hospital Kotor |
|  |  | Opšta Bolnica Meljine, Herceg Novi |
|  |  | JZU Dom Zdravlja Plav - Interno Odjeljenje |
|  |  | General Hospital Pljevlja |
|  |  | Opšta Bolnica Berane |
|  |  | Opšta Bolnica Bijelo Polje |
|  |  | Opšta Bolnica Danilo I, Cetinje |
|  |  | PZU OB.MELJINE |
|  | **Romania** |  |
|  |  | Spitalul Clinic de Urgenta, Bucaresti* |
|  |  | Spitalul Judetean, Baia Mare |
|  | **Serbia** |  |
|  |  | University of Belgrade, Clinical Center of Serbia, Belgrade* |
|  |  | Clinical Hospital Center, Zemun* |
|  |  | Zvezdara University Hospital Center |
|  |  | University Hospital Medical Center Bezanijska Kosa* |
|  |  | Hospital of Valjevo* |
|  |  | City Hospital, Mitrovica |
|  |  | Clinical Center Kragujevac* |
|  |  | General Hospital Cuprija |
|  |  | General Hospital Dr. Djordje Joanovic |
|  |  | Cardiology Clinic, Institute for Treatment and Rehabilitation, Nis* |
|  |  | General Hospital Jagodina |
|  |  | KBC Kosovska Mitrovica |
| **EMMACE 3X** | **United Kingdom** |  |
|  |  | Airedale NHS Foundation Trust |
|  |  | Basildon and Thurrock University Hospitals NHS Foundation Trust |
|  |  | Blackpool Teaching Hospitals NHS Foundation Trust |
|  |  | Bradford Teaching Hospitals NHS Foundation Trust* |
|  |  | Buckinghamshire Healthcare NHS Trust |
|  |  | Burton Hospitals NHS Foundation Trust |
|  |  | Calderdale and Huddersfield NHS Foundation Trust |
|  |  | Chesterfield Royal Hospital NHS Foundation Trust |
|  |  | City Hospitals Sunderland NHS Foundation Trust* |
|  |  | Colchester Hospital University NHS Foundation Trust |
|  |  | County Durham and Darlington NHS Foundation Trust |
|  |  | East Lancashire Hospitals NHS Trust |
|  |  | Frimley Park Hospital NHS Foundation Trust |
|  |  | Harrogate and District NHS Foundation Trust |
|  |  | Heatherwood and Wexham Park Hospitals NHS Foundation Trust |
|  |  | Hull and East Yorkshire Hospitals NHS Trust |
|  |  | Kettering General Hospital NHS Foundation Trust |
|  |  | Lancashire Teaching Hospitals NHS Foundation Trust |
|  |  | Leeds Teaching Hospitals NHS Trust |
|  |  | York Teaching Hospital NHS Foundation Trust |
|  |  | Medway NHS Foundation Trust |
|  |  | Mid Staffordshire NHS Foundation Trust |
|  |  | Mid Yorkshire Hospitals NHS Trust |
|  |  | Milton Keynes Hospital NHS Foundation Trust |
|  |  | Bagnall Newcastle Hospitals NHS Foundation Trust |
|  |  | North Cumbria University Hospitals NHS Trust |
|  |  | Northern Devon Healthcare NHS Trust |
|  |  | Northern Lincolnshire and Goole Hospitals NHS Foundation Trust |
|  |  | Northumbria Healthcare NHS Foundation Trust |
|  |  | Pennine Acute Hospitals NHS Trust |
|  |  | Peterborough and Stamford Hospitals Trust* |
|  |  | Plymouth Hospitals NHS Trust* |
|  |  | Portsmouth Hospitals NHS Trust |
|  |  | Royal Devon and Exeter NHS Foundation Trust |
|  |  | Scarborough and North East Yorkshire Health Care NHS Trust |
|  |  | Sherwood Forest Hospitals NHS Foundation Trust, |
|  |  | Shrewsbury and Telford Hospitals NHS Trust |
|  |  | South Devon Healthcare NHS Foundation Trust |
|  |  | South Tees Hospitals NHS Foundation Trust |
|  |  | Southend University Hospitals NHS Foundation Trust |
|  |  | Stockport NHS Foundation Trust |
|  |  | Taunton and Somerset NHS Foundation Trust |
|  |  | The Newcastle Upon Tyne Hospitals NHS Foundation Trust |
|  |  | University Hospital of Morecambe Bay NHS Foundation Trust* |
|  |  | University Hospitals of Leicester NHS Trust |
|  |  | West Middlesex University Hospital NHS Trust |
|  |  | Wigan and Leigh NHS Foundation Trust |
| Centers marked with * participated to the Odyssey Trial^3^ | | |

## **Risk factors definitions**

Smoking habits were self-reported. We defined current smokers as individuals who smoked 100 cigarettes in his or her lifetime and who smoked cigarettes, cigars, and cigarillos at the time of the index event. Everyday smokers or someday smokers were all included in this definition according to recommendations from the National Health Interview Survey.^4^ Participants who have smoked at least 100 cigarettes in their lifetime but who were not active smokers at the time of the index event were labelled as former smokers regardless of time since they quit. The remaining patients were classified as never smokers. Hypertension, hypercholesterolemia and diabetes were assessed by designation of medical history prior to admission in the database.

**Multiple Imputation using Chained Equation (MICE) algorithm**

Multiple Imputation using Chained Equation (MICE) algorithm is an efficient and popular method to fill in missing data where each missing value on some records is replaced by a value obtained from related cases in the whole set of records. Thus, imputation for clinical features was conducted using the chained equations across other features.^5^ More specifically, MICE algorithm sequentially imputes the missing values of clinical features based on both observed values and previously imputed values. This sequential imputation is conducted via chained equations.

We tried multiple imputations using the MICE algorithm for the initial analyses to address the uncertainty in the imputation process. More specifically, we generated multiple imputed datasets and check whether the conclusions are consistent across the different imputed datasets. If the conclusions are consistent across multiple imputed datasets, we use a single imputed dataset (by MICE algorithm) as the final dataset to report the results of statistical analyses in the paper.^5,6^

**Inverse Propensity Score Weighting Analysis**

We used Inverse Propensity Score Weighting (IPW) to balance the distribution of covariates between two patient groups. Note that we use Logistic Regression to estimate the propensity scores ({P}(Z=1 | x)) If *e* denotes the estimated propensity score (i.e. e=\hat{P}(Z=1 | x), where the patient x is included in patient group 1; then, 1-e = \hat{P}(Z=0 | x)), then the original sample is weighted by the following weights: Z/e+(1−Z)/ 1−e where Z represents the patient group. For instance, women (Z=1) are assigned a weight equal to the reciprocal of the propensity score (1/e), while men (Z=0) are assigned a weight equal to the reciprocal of one minus the propensity score (1/1-e). The weighting procedure for each sample balances the covariate distributions between two patient groups.^7^

Inverse probability of treatment weighting method can potentially result in unstable and biased estimates if some of the weights are very high. To avoid excessive weights, we compared results with other methods for handling confounding. We included probability of treatment variables in a multivariable model. We also used XGBoost, a decision-tree-based ensemble machine learning algorithm, as an alternative multivariable model for estimating the probability of treatment. Conclusions from theses analyses were the same as our current results. Further, we created a threshold for weights to avoid the impacts of the outliers (we use 0.01 as threshold). Therefore, the inverse probability of treatment weighting analyses presented in the current analysis were quite stable.

## **Computation of Relative Risk and its Confidence Interval**

In a two-group cohort study, the risk ratio (RR, also called relative risk), is usually applied to compare risks of a health event between two independent binomial populations that differ by a demographic characteristic (i.e. sex, age) or by the level of exposure to a specific drug or risk factor. In such types of studies, data can be summarized in a confusion matrix as follows:

|  | **Risk of Designated Outcome** | |  |
| --- | --- | --- | --- |
|  | **Yes** | **No** | **Total** |
| **Exposed** | a | b | a+b (*H_1_*) |
| **Unexposed** | c | d | c+d (*H_0_*) |
| **Total** | a+c | b+d |  |

Where *H_1_* and *H_0_* correspond to the total number of exposed and unexposed patients, respectively, whereas *a and c* represent the number of exposed and unexposed patients at risk for the designated outcome, respectively.

RR is defined as the ratio between the risk of outcome in exposed patients (*H_1_*) and the risk of outcome in unexposed patients (*H_0,_*) which can be summarized as:

$$RR=\frac{\left( \frac{a}{H_{1}} \right)}{\left( \frac{c}{H_{0}} \right)}$$

When applying this equation to an IPTW balanced population, $\frac{a}{H_{1}}$ will be assigned a weight equal to the reciprocal of the propensity score ($\frac{1}{e}$) and $\frac{c}{H_{0}}$ will be weighted by the reciprocal of one minus the propensity score ($\frac{1}{(1-e)}$).

In order to compute the lower and upper (1-α) confidence limit RR_L_ for RR, we operate in the assumption of log normal distribution.^8^ In particular, the variate $\log\frac{\left( \frac{a}{H_{1}} \right)}{\left( \frac{c}{H_{0}} \right)}$= $\log\frac{a}{H_{1}}- \log\frac{c}{H_{0}}$is approximately normally distributed with approximate mean log(RR) and estimated variance $\frac{1-\left( \frac{a}{H_{1}} \right)}{a}$ + $\frac{1-(\frac{c}{H_{0}})}{c}$ .

It follows that RR_L_ can be computed by solving the following equation:

$$\frac{\left[ log( \frac{\frac{a}{H_{1}}}{\frac{c}{H_{0}}})- \log({RR}_{L}) \right]}{\left[ \frac{1-\left( \frac{a}{H_{1}} \right)}{a} + \frac{1-(\frac{c}{H_{0}})}{c} \right]^{1/2}}=z_{1-\alpha}$$

Where $z_{1-\alpha}$, is the 100(1-α) percentage point of the N(O, 1) distribution

## **Comparison of means and prevalences in the weighted sample**

To evaluate the balance of the baseline covariate distributions between treatment and control groups, standardized difference (SD) is widely used in inverse probability of treatment weighting (IPTW) framework. For the baseline analysis, we use standard SD which is defined as follows: $\frac{m_{t}-m_{c}}{\sqrt{\frac{s_{t}^{2}+s_{c}^{2}}{2}}}$ for continuous variables and $\frac{m_{t}-m_{c}}{\sqrt{\frac{m_{t}(1-m_{t})+m_{c}(1-m_{c})}{2}}}$ for binary variable where $m_{t}, m_{c}$ are sample mean of the variables for treatment and control group, and $s_{t}^{2}, s_{c}^{2}$ are sample variance of the variables for treatment and control group, respectively. For IPTW analysis, we use weighted SD where $m_{t}, m_{c}$ are replaced to weighted sample mean of the variables for treatment and control group, and $s_{t}^{2}, s_{c}^{2}$ are replaced to weighted sample variance of the variables for treatment and control group, respectively. Weights are determined by the inverse probability of treatment received. In general, 0.1 is the reasonable threshold to determine whether two distributions are balanced (i.e., if SD > 0.1, the baseline covariate is imbalanced).^9^

**Interaction test**

The comparison of two estimated quantities, each with its standard error, is a general method that can be applied widely. We compared the risk ratios of 30-day mortality from two subgroups (women versus men) stratified by ACS type (STEMI vs NSTEMI). These measures were always analyzed on the log scale because the distributions of the log ratios tend to be those closer to normal than of the ratios themselves. If the estimates are *E*1 and *E*2 with standard errors SE(*E*1) and SE(*E*2), then the difference *d*=*E*1 - *E*2 has standard error SE(*d*)=Ö[SE(*E*1)2 + SE(*E*2)2] i.e., the square root of the sum of the squares of the separate standard errors. The ratio *z*=*d*/SE(*d*) gives a test of the null hypothesis that in the population the difference *d* is zero, by comparing the value of *z* to the standard normal distribution. The 95% confidence interval (CI) for the difference is *d*-1.96SE(*d*) to *d*+1.96SE(*d*).^10^

# **SUPPLEMENTAL RESULTS**

## **Interaction tests**

We tested **(Table S6)** whether there is a significant interaction between sex (women versus men) and country income (High Income Countries versus Middle Income Countries) in function of the outcome (30-day mortality)**.** We obtained the logs of the risk ratios and their confidence intervals (rows 2 and 4). As 95% confidence intervals were obtained as 1.96 standard errors (SE) either side of the estimate, the SE of each log relative risk was obtained by dividing the width of its confidence interval by 2×1.96 (row 6). The estimated difference in log relative risks was *d*=E1- E2= -0.37(row 7) and its standard error 0.13 (row 8). From these two values, we tested the interaction and estimated the ratio of the relative risks (with confidence interval). The test of interaction was the ratio of *d* to its standard error: z= -2.90, which gave a *P* value=0.002 when we referred it to a table of the normal distribution (row 10). The estimated interaction effect was exp =0,6895 (row 11). The confidence interval for this effect was -0.62 to -0.12 on the log scale (row 9). Transforming back to the relative risk scale, we got 0.54 to 0.89 (row 12). We repeated the analysis according to use of revascularization **(Table S13 and S15)**. The analysis was repeated to assess potential interaction between sex and country-level income (High Income vs Middle Income) or type of MI (STEMI vs NSTEMI) in function of the outcome (30-day mortality) **(Tables S8, S10, S17 and S19).**

## **Figure S1.** Rates of STEMI patients presenting <2 hours from symptoms onset by sex and income categories


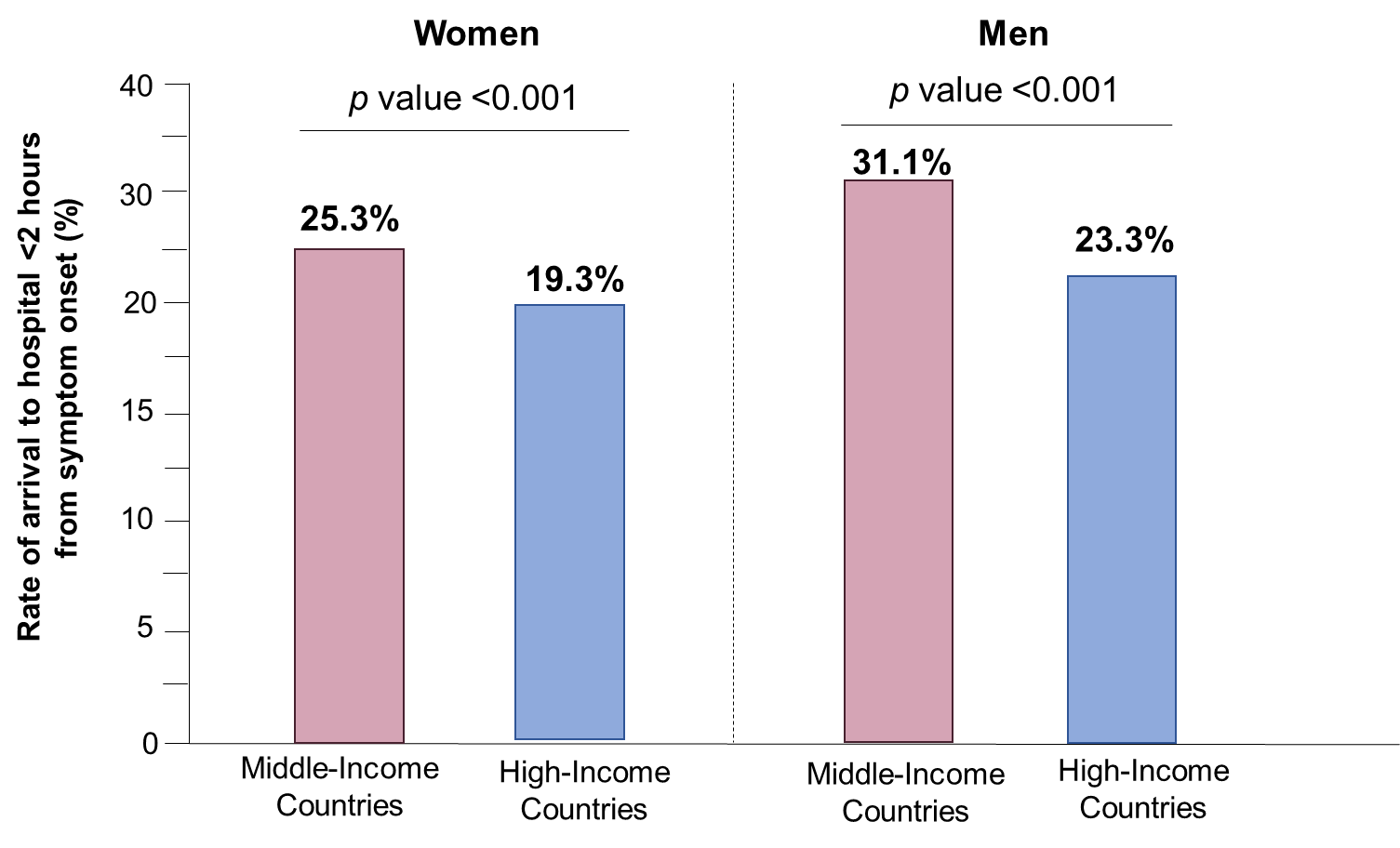


| **Table S1.** Rate of missing values for covariates used for inverse probability weighting models | |
| --- | --- |
| **Variable name** | **Rate of missing values (%)** |
| Age, years | - |
| **Cardiovascular risk factors** | |
| Diabetes | 3.1 |
| Hypertension | 2.4 |
| Hypercholesterolemia | 10.7 |
| Current smokers | 5.3 |
| Former smokers | 5.3 |
| **Clinical history of CHD** | |
| Prior angina pectoris | 0.2 |
| Prior myocardial infarction | 0.2 |
| Prior PCI | 0.2 |
| Prior CABG | 0.2 |
| **Clinical history of CVD** | |
| Peripheral artery disease | 0.9 |
| **Clinical presentation** |  |
| STEMI | - |
| SBP at admission, mmHg | 18.0 |
| HR at admission, bpm | 18.2 |
| Serum creatinine at admission, mg/dL | 18.0 |
| **Revascularization procedures** |  |
| PCI | 0.8 |
| Data are presented as percentages (%)  Abbreviations: CABG=coronary artery bypass graft; CHD=coronary heart disease; CVD=cardiovascular disease; PCI=percutaneous coronary intervention. | |

| **Table S2.** Inverse probability weighting: clinical factors and outcomes stratified by sex and economic status in patients with myocardial infarction | | | | | | |
| --- | --- | --- | --- | --- | --- | --- |
|  | **High Income countries** | | | **Middle Income countries** | | |
| **Characteristics** | **Women**  **(n=2,477)** | **Men**  **(n=5,989)** | **Standardized difference** | **Women**  **(n=4,191)** | **Men**  **(n=9,429)** | **Standardized difference** |
| Age, years | 64.6 ± 12.4 | 64.7 ± 12.4 | -0.01 | 61.8 ± 12.2 | 61.9 ± 11.7 | -0.01 |
| **Cardiovascular risk factors** |  |  |  |  |  |  |
| Diabetes, % | 21.7 | 21.9 | -0.01 | 26.2 | 25.3 | 0.02 |
| Hypertension, % | 59.4 | 60.3 | -0.02 | 70.1 | 69.0 | 0.02 |
| Hypercholesterolemia, % | 41.2 | 40.2 | 0.02 | 40.7 | 41.7 | -0.02 |
| Current smokers, % | 35.6 | 35.7 | -0.001 | 42.4 | 42.3 | 0.0004 |
| Former smokers, % | 26.0 | 24.2 | 0.04 | 5.3 | 5.3 | 0.002 |
| **Clinical history of CHD** |  |  |  |  |  |  |
| Prior angina pectoris, % | 15.3 | 14.4 | 0.03 | 15.4 | 15.7 | -0.01 |
| Prior MI, % | 16.5 | 15.5 | 0.03 | 13.8 | 14.5 | -0.02 |
| Prior PCI, % | 9.5 | 8.4 | 0.04 | 11.5 | 11.9 | -0.01 |
| Prior CABG, % | 4.0 | 3.7 | 0.02 | 1.9 | 2.0 | -0.002 |
| **Clinical history of CVD** |  |  |  |  |  |  |
| Peripheral artery disease, % | 4.5 | 4.3 | 0.01 | 1.5 | 1.5 | -0.001 |
| **Clinical presentation at admission** | |  |  |  |  |  |
| Systolic blood pressure, mmHg | 138.9 ± 28.4 | 138.5 ± 31.3 | 0.01 | 139.8 ± 29.1 | 139.7 ± 27.2 | 0.003 |
| Heart rate, bpm | 79.4 ± 24.2 | 79.3 ± 20.3 | 0.001 | 82.0 ± 20.7 | 81.6 ± 18.8 | 0.02 |
| Serum creatinine levels, mg/dL | 1.5 ± 1.2 | 1.1 ± 0.7 | 0.09 | 1.3 ± 1.2 | 1.1 ± 0.9 | 0.06 |
| **Outcomes** |  |  | ***p* value** |  |  | ***p* value** |
| 30-day mortality, % | 5.5 | 4.2 | 0.02 | 10.3 | 5.7 | <0.0001 |
| Risk Ratio (95% CI) | 1.31 (1.06 – 1.63) | | 0.01 | 1.90 (1.67 – 2.17) | | <0.0001 |
| Data are presented as percentages (%) or mean ± standard deviation, unless otherwise specified.  Abbreviations: bpm=beats per minute; CABG=coronary artery bypass graft; CHD=coronary heart disease; CVD=cardiovascular disease; PAD=peripheral artery disease; PCI=percutaneous coronary intervention. | | | | | | |

| **Table S3.** General logistic regression and regression coefficients in the propensity score model in the overall study population (women versus men) | | | | |
| --- | --- | --- | --- | --- |
|  | $\boldsymbol{\beta}$ | **SE** | **T statistics** | ***p* value** |
| **Constant term (α)** | -2.0898 | 0.2785 | -7.5032 | <0.0001 |
| Age | 0.3452 | 0.0666 | 5.1822 | <0.0001 |
| Diabetes | 0.2610 | 0.0438 | 5.9522 | <0.0001 |
| Hypertension | 0.3546 | 0.0663 | 5.3435 | <0.0001 |
| Hypercholesterolemia | 0.0582 | 0.0354 | 1.6439 | 0.1001 |
| Current smokers | -0.5429 | 0.1031 | -5.2686 | <0.0001 |
| Former smokers | -0.9726 | 0.2401 | -4.0514 | <0.0001 |
| Prior angina pectoris | 0.1094 | 0.0654 | 1.6724 | 0.0944 |
| Prior MI | -0.1151 | 0.0553 | -2.0794 | 0.0375 |
| Prior PCI | -0.1606 | 0.0837 | -1.7195 | 0.0551 |
| Prior CABG | -0.4098 | 0.2770 | -1.4794 | 0.1390 |
| Peripheral artery disease | -0.0810 | 0.5075 | -1.5964 | 0.1104 |
| SPB at admission mm Hg | -0.0026 | 0.0010 | -2.4189 | 0.0155 |
| HR at admission bpm | 0.0012 | 0.0009 | 1.3568 | 0.1748 |
| Serum creatinine levels at admission mg/dL | -0.5512 | 0.0953 | -5.7839 | <0.0001 |
| Optimized regression coefficient (ß) and constant term(α) for the logistic regression.  Abbreviations: CABG= coronary artery bypass graft; HR=heart rate; MI=myocardial infarction; PCI=percutaneous coronary intervention; SBP=systolic blood pressure | | | | |

| **Table S4.** General logistic regression and regression coefficients in the propensity score model in women (high income countries vs middle income countries) | | | | |
| --- | --- | --- | --- | --- |
|  | $\boldsymbol{\beta}$ | **SE** | **T statistics** | ***p* value** |
| **Constant term (α)** | -1.5983 | 1.3155 | -1.2149 | 0.2243 |
| Age, years | 0.2243 | 0.1356 | 1.6547 | 0.0979 |
| Diabetes | -0.3227 | 0.1381 | -2.3366 | 0.0194 |
| Hypertension | -0.5778 | 0.3770 | -1.5323 | 0.1254 |
| Hypercholesterolemia | 0.0840 | 0.3199 | 0.2625 | 0.7929 |
| Current smokers | 0.1429 | 0.3432 | 0.4163 | 0.6772 |
| Former smokers | 2.0805 | 0.6451 | 3.2251 | 0.0012 |
| Prior angina pectoris | -0.7072 | 0.4933 | -1.4336 | 0.1516 |
| Prior MI | 0.0881 | 0.2386 | 0.3691 | 0.7120 |
| Prior PCI | -0.4970 | 0.4239 | -1.1724 | 0.2410 |
| Prior CABG | 0.8169 | 0.2746 | 2.9747 | 0.0029 |
| Peripheral artery disease | 0.9779 | 0.4250 | 2.3006 | 0.0214 |
| SPB at admission, mm Hg | 0.0004 | 0.0071 | 0.0589 | 0.9530 |
| HR at admission, bpm | -0.0043 | 0.0026 | -1.6627 | 0.9636 |
| Serum creatinine levels at admission, mg/dL | 0.1142 | 0.0718 | 1.5905 | 0.1117 |
| Optimized regression coefficient (ß) and constant term(α) for the logistic regression.  Abbreviations: CABG=coronary artery bypass graft; HR=heart rate; MI=myocardial infarction; PCI=percutaneous coronary intervention; SBP=systolic blood pressure | | | | |

| **Table S5.** General logistic regression and regression coefficients in the propensity score model in men (high income countries vs middle income countries) | | | | |
| --- | --- | --- | --- | --- |
|  | $\boldsymbol{\beta}$ | **SE** | **T statistics** | ***p* value** |
| **Constant term (α)** | -1.3061 | 1.1040 | -1.1831 | 0.2368 |
| Age, years | 0.1943 | 0.1134 | 1.7126 | 0.0868 |
| Diabetes | -0.1703 | 0.1422 | -1.1975 | 0.2311 |
| Hypertension | -0.4589 | 0.2952 | -1.5539 | 0.1202 |
| Hypercholesterolemia | 0.0219 | 0.2977 | 0.0734 | 0.9415 |
| Current smokers | 0.1969 | 0.1190 | 1.6545 | 0.0980 |
| Former smokers | 1.7160 | 0.4092 | 4.1933 | <0.0001 |
| Prior angina pectoris | -0.1663 | 0.4511 | -0.3687 | 0.7124 |
| Prior MI | 0.0525 | 0.2139 | 0.2456 | 0.8060 |
| Prior PCI | -0.4387 | 0.2870 | -1.5283 | 0.1264 |
| Prior CABG | 0.2487 | 0.3484 | 0.7138 | 0.4753 |
| Peripheral artery disease | 0.9813 | 0.4264 | 2.3013 | 0.0213 |
| SPB at admission, mmHg | -0.0001 | 0.0056 | -0.0091 | 0.9927 |
| HR at admission, bpm | -0.0064 | 0.0026 | -2.3748 | 0.0175 |
| Serum creatinine levels at admission, mg/dL | 0.0296 | 0.1447 | 0.2044 | 0.8380 |
| Optimized regression coefficient (ß) and constant term(α) for the logistic regression.  Abbreviations: CABG=coronary artery bypass graft; HR=heart rate; MI=myocardial infarction; PCI=percutaneous coronary intervention; SBP=systolic blood pressure | | | | |

| **Table S6.** Interaction test: calculations for comparing two estimated risk ratios (women vs men) for 30-day mortality by inverse probability weighting: high-income versus middle-income countries | | | |
| --- | --- | --- | --- |
|  |  | **Group 1**  **[High income countries]**  **(n = 8,466)** | **Group 2**  **[Middle income countries]**  **(n = 13,620)** |
| **1** | **RR** | 1.31 | 1.90 |
| **2** | **log RR** | 0.27 | 0.64 |
| **3** | **95% CI for RR** | 1.06-1.63 | 1.67-2.17 |
| **4** | **95% CI for log RR** | 0.06-0.49 | 0.51-0.77 |
| **5** | **Width of CI** | 0.43 | 0.26 |
| **6** | **SE (=width / (2*1.96))** | 0.11 | 0.07 |
| **Difference between log risk ratios** | | | |
| **7** | **d (=**$\boldsymbol{E}_{\boldsymbol{1}}\boldsymbol{-}\boldsymbol{E}_{\boldsymbol{2}}$**)** | -0.37 | |
| **8** | **SE (d)** | 0.13 | |
| **9** | **CI (d)** | -0.62- (-0.12) | |
| **10** | **Test of Interaction** | -2.90 **(*p* value:0.002)** | |
| **Ratio of risk ratios** | | | |
| **11** | **RRR (=exp(d) )** | 0.69 | |
| **12** | **CI (RRR)** | 0.54-0.89 | |

| **Table S7.** Inverse probability weighting: clinical factors and outcomes stratified by sex and economic status income in STEMI patients | | | | | | |
| --- | --- | --- | --- | --- | --- | --- |
|  | **High Income countries** | | | **Middle Income countries** | | |
| **Characteristics** | **Women**  **(n=1,426)** | **Men**  **(n=3,465)** | **Standardized difference** | **Women**  **(n=2,884)** | **Men**  **(n=6,690)** | **Standardized difference** |
| Age, years | 62.5 ± 13.0 | 62.9 ± 12.4 | -0.03 | 61.1 ± 12.0 | 61.4 ± 11.7 | -0.02 |
| **Cardiovascular risk factors** |  |  |  |  |  |  |
| Diabetes, % | 18.8 | 20.5 | -0.04 | 23.0 | 23.7 | -0.02 |
| Hypertension, % | 56.5 | 57.0 | -0.01 | 68.9 | 66.5 | 0.05 |
| Hypercholesterolemia, % | 35.1 | 37.5 | -0.05 | 41.1 | 42.2 | -0.02 |
| Current smokers, % | 42.2 | 42.2 | 0.0001 | 47.2 | 45.4 | 0.04 |
| Former smokers, % | 18.8 | 18.7 | 0.002 | 5.1 | 5.4 | -0.02 |
| **Clinical history of CHD** |  |  |  |  |  |  |
| Prior angina pectoris, % | 7.9 | 8.2 | -0.01 | 12.0 | 13.0 | -0.03 |
| Prior MI, % | 10.5 | 10.0 | 0.02 | 11.0 | 11.7 | -0.02 |
| Prior PCI, % | 4.8 | 4.8 | -0.0002 | 11.5 | 11.9 | -0.01 |
| Prior CABG, % | 4.1 | 2.5 | 0.09 | 1.3 | 1.3 | 0.001 |
| **Clinical history of CVD** |  |  |  |  |  |  |
| Peripheral artery disease, % | 3.5 | 3.6 | -0.01 | 1.5 | 1.6 | -0.01 |
| **Clinical presentation at admission** | |  |  |  |  |  |
| Systolic blood pressure, mmHg | 136.0 ± 29.6 | 134.9 ± 27.0 | 0.04 | 138.3 ± 29.0 | 137.7 ± 27.5 | 0.02 |
| Heart rate, bpm | 79.6 ± 21.3 | 79.0 ± 19.9 | 0.03 | 81.2 ± 21.5 | 81.1 ± 18.9 | 0.01 |
| Serum creatinine levels, mg/dL | 1.3 ± 1.2 | 1.1 ± 0.9 | 0.08 | 1.4 ± 1.4 | 1.2 ± 1.0 | 0.07 |
| **Outcomes** |  |  | ***p* value** |  |  | ***p* value** |
| 30-day mortality, % | 6.8 | 5.1 | 0.03 | 12.4 | 5.8 | <0.0001 |
| Risk Ratio (95% CI) | 1.36 (1.05 – 1.75) | | 0.02 | 2.30 (1.98 – 2.68) | | <0.0001 |
| Data are presented as percentages (%) or mean ± standard deviation, unless otherwise specified.  Abbreviations: bpm=beats per minute; CABG=coronary artery bypass graft; CHD=coronary heart disease; CVD=cardiovascular disease; PAD=peripheral artery disease; PCI=percutaneous coronary intervention; STEMI=ST-segment elevation myocardial infarction. | | | | | | |

| **Table S8.** Interaction test: calculations for comparing two estimated risk ratios (women vs men) for 30-day mortality by inverse probability weighting: high-income countries versus middle-income countries in STEMI patients | | | |
| --- | --- | --- | --- |
|  |  | **Group 1**  **[High-income countries]**  **(n = 8,466)** | **Group 2**  **[Middle-income countries]**  **(n = 13,620)** |
| **1** | **RR** | 1.36 | 2.30 |
| **2** | **log RR** | 0.31 | 0.83 |
| **3** | **95% CI for RR** | (1.05 – 1.75) | (1.98 – 2.68) |
| **4** | **95% CI for log RR** | 0.05-0.56 | 0.68-0.99 |
| **5** | **Width of CI** | 0.51 | 0.30 |
| **6** | **SE (=width / (2*1.96))** | 0.13 | 0.08 |
|  | | | |
| **7** | **d (=**$\boldsymbol{E}_{\boldsymbol{1}}\boldsymbol{-}\boldsymbol{E}_{\boldsymbol{2}}$**)** | **-0.52** | |
| **8** | **SE (d)** | **0.15** | |
| **9** | **CI (d)** | -0.82-(-0.22) | |
| **10** | **Test of Interaction** | -3.47 (***p* value<0.001**) | |
|  | | | |
| **11** | **RRR (=exp(d) )** | 0.59 | |
| **12** | **CI (RRR)** | 0.44-0.80 | |

| **Table S9.** Inverse probability weighting: clinical factors and outcomes stratified by sex and economic status in NSTEMI patients | | | | | | |
| --- | --- | --- | --- | --- | --- | --- |
|  | **High-Income countries** | | | **Middle-Income countries** | | |
| **Characteristics** | **Women**  **(n=1,049)** | **Men**  **(n=2,524)** | **Standardized difference** | **Women**  **(n=1,307)** | **Men**  **(n=2,739)** | **Standardized difference** |
| Age, years | 67.1 ± 11.9 | 67.2 ± 11.8 | -0.01 | 62.9 ± 12.2 | 63.0 ± 11.6 | -0.01 |
| **Cardiovascular risk factors** |  |  |  |  |  |  |
| Diabetes, % | 23.3 | 23.7 | -0.01 | 30.1 | 29.0 | 0.02 |
| Hypertension, % | 63.9 | 64.7 | -0.01 | 74.8 | 74.8 | 0.0001 |
| Hypercholesterolemia, % | 43.6 | 43.8 | -0.003 | 40.1 | 40.8 | -0.01 |
| Current smokers, % | 26.8 | 26.8 | 0.0003 | 34.5 | 35.1 | -0.01 |
| Former smokers, % | 32.3 | 31.7 | 0.01 | 5.2 | 4.9 | 0.01 |
| **Clinical history of CHD** |  |  |  |  |  |  |
| Prior angina pectoris, % | 23.4 | 22.8 | 0.01 | 21.9 | 22.3 | -0.01 |
| Prior MI, % | 23.5 | 23.1 | 0.01 | 20.2 | 21.2 | -0.03 |
| Prior PCI, % | 13.7 | 13.3 | 0.01 | 11.4 | 11.7 | -0.01 |
| Prior CABG, % | 6.9 | 6.8 | 0.006 | 3.3 | 3.6 | 0.01 |
| **Clinical history of CVD** |  |  |  |  |  |  |
| Peripheral artery disease, % | 5.4 | 5.2 | 0.007 | 1.3 | 1.2 | 0.01 |
| **Clinical presentation at admission** | |  |  |  |  |  |
| Systolic blood pressure, mmHg | 143.0 ± 27.1 | 143.5 ± 35.7 | -0.02 | 144.3 ± 27.8 | 144.5 ± 25.9 | -0.009 |
| Heart rate, bpm | 79.8 ± 23.2 | 79.9 ± 20.9 | 0.0004 | 83.1 ± 19.9 | 82.8 ± 18.8 | 0.01 |
| Serum creatinine levels, mg/dL | 1.2 ± 1.1 | 1.1 ± 0.9 | 0.05 | 1.2 ± 1.1 | 1.1 ± 0.9 | 0.06 |
| **Outcomes**, % |  |  | ***p* value** |  |  | ***p* value** |
| 30-day mortality, % | 4.1 | 3.0 | 0.13 | 7.0 | 5.5 | 0.07 |
| Risk Ratio (95% CI) | 1.36 (0.93 – 2.00) | | 0.11 | 1.30 (0.99 – 1.70) | | 0.06 |
| Data are presented as percentages (%) or mean ± standard deviation, unless otherwise specified.  Abbreviations: bpm=beats per minute; CABG=coronary artery bypass graft; CHD=coronary heart disease; CVD=cardiovascular disease; NSTEMI=non-ST segment elevation myocardial infarction; PAD=peripheral artery disease; PCI=percutaneous coronary intervention | | | | | | |

| **Table S10.** Interaction test: calculations for comparing two estimated risk ratios (women vs men) for 30-day mortality by inverse probability weighting: high-income countries versus middle-income countries in NSTEMI patients | | | |
| --- | --- | --- | --- |
|  |  | **Group 1**  **[High-income countries]**  **(n = 8,466)** | **Group 2**  **[Middle-income countries]**  **(n = 13,620)** |
| **1** | **RR** | 1.36 | 1.30 |
| **2** | **log RR** | 0.31 | 0.26 |
| **3** | **95% CI for RR** | (0.93 – 2.00) | (0.99 – 1.70) |
| **4** | **95% CI for log RR** | -0.07-0.69 | -0.01-0.53 |
| **5** | **Width of CI** | 0.77 | 0.54 |
| **6** | **SE (=width / (2*1.96))** | 0.20 | 0.14 |
|  | | | |
| **7** | **d (=**$\boldsymbol{E}_{\boldsymbol{1}}\boldsymbol{-}\boldsymbol{E}_{\boldsymbol{2}}$**)** | **0.05** | |
| **8** | **SE (d)** | **0.24** | |
| **9** | **CI (d)** | -0.42-0.51 | |
| **10** | **Test of Interaction** | 0.19 (***p* value**: 0.43) | |
|  | | | |
| **11** | **RRR (=exp(d) )** | 1.05 | |
| **12** | **CI (RRR)** | 0.65-1.67 | |

| **Table S11.** Inverse probability weighting: clinical factors and outcomes stratified by sex and economic status in patients with myocardial infarction undergoing revascularization | | | | | | |
| --- | --- | --- | --- | --- | --- | --- |
|  | **High-income countries** | | | **Middle-income countries** | | |
| **Characteristics** | **Women**  **(n=1,384)** | **Men**  **(n=3,667)** | **Standardized difference** | **Women**  **(n=3,019)** | **Men**  **(n=7,540)** | **Standardized difference** |
| Age, years | 63.5 ± 11.9 | 63.3 ± 12.0 | 0.01 | 60.6 ± 11.9 | 60.7 ± 11.3 | -0.01 |
| **Cardiovascular risk factors** |  |  |  |  |  |  |
| Diabetes, % | 20.6 | 19.9 | 0.02 | 23.9 | 23.4 | 0.01 |
| Hypertension, % | 60.5 | 60.7 | -0.005 | 68.1 | 67.8 | 0.007 |
| Hypercholesterolemia, % | 43.0 | 44.4 | -0.03 | 41.4 | 41.6 | -0.004 |
| Current smokers, % | 40.0 | 41.1 | -0.02 | 45.8 | 46.1 | -0.006 |
| Former smokers, % | 22.5 | 22.3 | 0.006 | 5.4 | 5.3 | 0.005 |
| **Clinical history of CHD** |  |  |  |  |  |  |
| Chronic stable angina, % | 14.0 | 13.7 | 0.009 | 11.4 | 11.2 | 0.005 |
| Prior MI, % | 13.2 | 12.7 | 0.01 | 13.0 | 12.8 | 0.005 |
| Prior PCI, % | 8.8 | 8.8 | 0.0009 | 11.8 | 11.6 | 0.005 |
| Prior CABG, % | 2.9 | 2.9 | -0.004 | 1.0 | 1.0 | -0.001 |
| **Clinical history of CVD** |  |  |  |  |  |  |
| Peripheral arterial disease, % | 3.8 | 3.5 | 0.02 | 1.3 | 1.2 | 0.004 |
| **Clinical presentation on admission** | |  |  |  |  |  |
| Systolic blood pressure, mmHg | 138.3 ± 28.7 | 138.1 ± 33.2 | 0.005 | 140.7 ± 28.9 | 140.4 ± 27.8 | 0.01 |
| Heart rate, bpm | 78.0 ± 19.1 | 78.0 ± 18.6 | -0.003 | 80.7 ± 18.4 | 80.9 ± 17.8 | -0.02 |
| Serum creatinine levels, mg/dL | 1.1 ± 0.9 | 1.0 ± 0.6 | 0.06 | 1.1 ± 0.7 | 1.0 ± 0.6 | 0.02 |
| **Outcomes** |  |  | ***p* value** |  |  | ***p* value** |
| 30-day mortality, % | 4.3 | 2.3 | 0.001 | 6.9 | 3.8 | <0.0001 |
| Risk Ratio (95% CI) | 1.89 (1.35 – 2.66) | | 0.0002 | 1.89 (1.58 – 2.28) | | <0.0001 |
| Data are presented as percentages (%) or means (standard deviation), unless otherwise specified.  Abbreviations: bpm=beats per minute; CABG=coronary artery bypass graft; CHD=coronary heart disease; CVD=cardiovascular disease; PAD= peripheral artery disease; PCI=percutaneous coronary intervention | | | | | | |

| **Table S12.** Inverse probability weighting: clinical factors and outcomes stratified by economic status and revascularization choice in women with myocardial infarction. | | | | | | |
| --- | --- | --- | --- | --- | --- | --- |
|  | **Middle-Income Countries** | | | **High-income countries** | | |
| **Characteristics** | **PCI**  **(n=3024)** | **No PCI**  **(n=1168)** | **Standardized difference** | **PCI**  **(n=1388)** | **No PCI**  **(n=1089)** | **Standardized difference** |
| Age, years | 65.7 ± 11.2 | 65.4 ± 12.4) | 0.03 | 67.8 ± 11.7 | 67.7 ± 12.7 | 0.009 |
| **Cardiovascular risk factors** |  |  |  |  |  |  |
| Diabetes, % | 31.6 | 31.3 | 0.007 | 25.2 | 24.6 | 0.01 |
| Hypertension, % | 77.6 | 77.8 | -0.004 | 67.6 | 66.9 | 0.01 |
| Hypercholesterolemia, % | 42.9 | 43.1 | -0.005 | 41.9 | 42.4 | -0.01 |
| Current smokers, % | 31.1 | 30.7 | 0.01 | 27.2 | 27.5 | -0.006 |
| Former smokers, % | 2.7 | 2.9 | -0.01 | 17.5 | 17.6 | -0.005 |
| **Clinical history of CHD** |  |  |  |  |  |  |
| Prior angina pectoris, % | 18.7 | 18.8 | -0.004 | 14.0 | 14.1 | -0.004 |
| Prior MI, % | 13.2 | 13.0 | 0.004 | 15.3 | 14.9 | 0.01 |
| Prior PCI, % | 11.3 | 11.1 | 0.005 | 7.8 | 7.9 | -0.007 |
| Prior CABG, % | 1.2 | 1.3 | -0.01 | 3.6 | 3.5 | 0.006 |
| **Clinical history of CVD** |  |  |  |  |  |  |
| Peripheral artery disease, % | 1.6 | 1.6 | -0.001 | 4.0 | 4.0 | -0.004 |
| **Clinical presentation at admission** | |  |  |  |  |  |
| Systolic blood pressure, mmHg | 139.4 ± 28.3 | 139.9 ± 28.5 | -0.02 | 138.2 ± 28.6 | 138.3 ± 29.4 | -0.0002 |
| Heart rate, bpm | 82.1 ± 19.2 | 82.2 ± 20.0 | -0.006 | 79.9 ± 20.4 | 79.9 ± 22.4 | -0.004 |
| Serum creatinine levels, mg/dL | 1.0 ± 0.6 | 1.0 ± 0.5 | -0.03 | 1.1 ± 0.9 | 1.1 ± 0.6 | 0.03 |
| **Outcomes** |  |  | ***p* value** |  |  | ***p* value** |
| 30-day mortality, % | 8.7 | 12.7 | 0.0003 | 5.5 | 8.6 | 0.003 |
| Risk Ratio (95% CI) | 0.66 (0.53 – 0.82) | | 0.0001 | 0.61 (0.45 – 0.84) | | 0.002 |
| Data are presented as percentages (%) or mean ± standard deviation, unless otherwise specified.  Abbreviations: bpm=beats per minute; CABG=coronary artery bypass graft; CHD=coronary heart disease; CVD=cardiovascular disease; PAD=peripheral artery disease; PCI=percutaneous coronary intervention. | | | | | | |

| **Table S13.** Interaction test: calculations for comparing two estimated risk ratios (No PCI versus PCI) for 30-day mortality by inverse probability weighting: Women | | | |
| --- | --- | --- | --- |
|  |  | **Group 1**  **[Middle-income countries]**  **(n=4,192)** | **Group 2**  **[High-income countries]**  **(n=2,477)** |
| **1** | **RR** | 0.66 | 0.61 |
| **2** | **log RR** | -0.4155 | -0.4943 |
| **3** | **95% CI for RR** | 0.53 – 0.82 | 0.45 – 0.84 |
| **4** | **95% CI for log RR** | -0.6349 - -0.1985 | -0.7985 - -0.1744 |
| **5** | **Width of CI** | 0.4361 | 0.6241 |
| **6** | **SE (=width / (2*1.96))** | 0.1113 | 0.1592 |
| **Difference between log risk ratios** | | | |
| **7** | **d (=**$\boldsymbol{E}_{\boldsymbol{1}}\boldsymbol{-}\boldsymbol{E}_{\boldsymbol{2}}$**)** | **0.0788** | |
| **8** | **SE (d)** | **0.1942** | |
| **9** | **CI (d)** | -0.3018 – 0.4594 | |
| **10** | **Test of Interaction** | 0.4058 (***p* value: 0.34)** | |
| **Ratio of risk ratios** | | | |
| **11** | **RRR (=exp(d) )** | 1.0820 | |
| **12** | **CI (RRR)** | 0.7395 – 1.5831 | |

| **Table S14.** Inverse probability weighting: clinical factors and outcomes stratified by economic status and revascularization choice in men with myocardial infarction | | | | | | |
| --- | --- | --- | --- | --- | --- | --- |
|  | **Middle-Income Countries** | | | **High-income countries** | | |
| **Characteristics** | **PCI**  **(n=7,540)** | **No PCI**  **(n=1,889)** | **Standardized difference** | **PCI**  **(n=3,667)** | **No PCI**  **(n=2,322)** | **Standardized difference** |
| Age, years | 60.2 ± 11.3 | 59.5 ± 12.8 | 0.06 | 63.4 ± 12.0 | 63.4 ± 12.7 | -0.004 |
| **Cardiovascular risk factors** |  |  |  |  |  |  |
| Diabetes, % | 22.5 | 21.3 | 0.03 | 20.7 | 21.0 | -0.006 |
| Hypertension, % | 65.3 | 64.8 | 0.01 | 58.2 | 58.5 | -0.007 |
| Hypercholesterolemia, % | 41.5 | 42.0 | -0.01 | 40.2 | 40.4 | -0.003 |
| Current smokers, % | 47.3 | 48.6 | -0.03 | 38.8 | 38.7 | 0.001 |
| Former smokers, % | 6.4 | 6.2 | 0.008 | 27.2 | 27.2 | 0.001 |
| **Clinical history of CHD** |  |  |  |  |  |  |
| Prior angina pectoris, % | 14.5 | 14.1 | 0.01 | 14.8 | 14.8 | 0.0003 |
| Prior MI, % | 15.0 | 14.8 | 0.006 | 15.9 | 15.8 | 0.005 |
| Prior PCI, % | 12.4 | 13.1 | -0.02 | 8.8 | 8.7 | 0.003 |
| Prior CABG, % | 2.4 | 2.3 | 0.003 | 3.9 | 3.9 | -0.0004 |
| **Clinical history of CVD** |  |  |  |  |  |  |
| Peripheral artery disease, % | 1.5 | 1.4 | 0.004 | 4.3 | 4.4 | -0.003 |
| **Clinical presentation at admission** | |  |  |  |  |  |
| Systolic blood pressure, mmHg | 140.0 ± 27.1 | 140.5 ± 27.3 | -0.02 | 141.1 ± 68.5 | 139.3 ± 27.6 | 0.04 |
| Heart rate, bpm | 81.3 ± 18.2 | 81.2 ± 20.2 | 0.02 | 78.9 (9.3) | 78.9 ± 20.4 | 0.003 |
| Serum creatinine levels, mg/dL | 1.1 ± 1.1 | 1.1 ± 0.7 | 0.02 | 1.1 ± 0.8 | 1.1 ± 0.7 | 0.01 |
| **Outcomes** |  |  | ***p* value** |  |  | ***p* value** |
| 30-day mortality, % | 4.3 | 7.8 | <0.0001 | 2.6 | 5.1 | <0.0001 |
| Risk Ratio (95% CI) | 0.53 (0.43 – 0.64) | | <0.0001 | 0.50 (0.38 – 0.66) | | <0.0001 |
| Data are presented as percentages (%) or mean ± standard deviation, unless otherwise specified.  Abbreviations: bpm=beats per minute; CABG=coronary artery bypass graft; CHD=coronary heart disease; CVD=cardiovascular disease; PAD=peripheral artery disease; PCI=percutaneous coronary intervention. | | | | | | |

| **Table S15.** Interaction test: calculations for comparing two estimated risk ratios (No PCI versus PCI) for 30-day mortality by inverse probability weighting: Men | | | |
| --- | --- | --- | --- |
|  |  | **Group 1**  **[High-income countries]**  **(n=9,429)** | **Group 2**  **[Middle-income countries]**  **(n=5,989)** |
| **1** | **RR** | 0.53 | 0.50 |
| **2** | **log RR** | -0.6349 | -0.6931 |
| **3** | **95% CI for RR** | 0.43 – 0.64 | 0.38 – 0.66 |
| **4** | **95% CI for log RR** | -0.8440 - -0.4463 | -0.9676 - -0.4155 |
| **5** | **Width of CI** | 0.3977 | 0.5521 |
| **6** | **SE (=width / (2*1.96))** | 0.1015 | 0.1408 |
| **Difference between log risk ratios** | | | |
| **7** | **d (=**$\boldsymbol{E}_{\boldsymbol{1}}\boldsymbol{-}\boldsymbol{E}_{\boldsymbol{2}}$**)** | **0.0582** | |
| **8** | **SE (d)** | **0.1736** | |
| **9** | **CI (d)** | -0.2821 – 0.3985 | |
| **10** | **Test of Interaction** | 0.3353 (***p* value: 0.36**) | |
| **Ratio of risk ratios** | | | |
| **11** | **RRR (=exp(d) )** | 1.0599 | |
| **12** | **CI (RRR)** | 0.7542 – 1.4896 | |

| **Table S16.** Inverse probability weighting: clinical factors and outcomes stratified by sex and economic status in STEMI patients undergoing revascularization | | | | | | |
| --- | --- | --- | --- | --- | --- | --- |
|  | **High-Income countries** | | | **Middle-income countries** | | |
| **Characteristics** | **Women**  **(n=885)** | **Men**  **(n=2,381)** | **Standardized difference** | **Women**  **(n=2,319)** | **Men**  **(n=5,750)** | **Standardized difference** |
| Age, years | 62.6 ± 12.3 | 62.2 ± 12.0 | 0.03 | 60.8 ± 11.9 | 60.6 ± 11.4 | 0.02 |
| **Cardiovascular risk factors** |  |  |  |  |  |  |
| Diabetes, % | 19.5 | 18.6 | 0.02 | 23.4 | 22.3 | 0.03 |
| Hypertension, % | 57.0 | 57.5 | -0.01 | 67.1 | 66.4 | 0.01 |
| Hypercholesterolemia, % | 40.6 | 41.3 | -0.01 | 42.6 | 42.6 | 0.0003 |
| Current smokers, % | 45.8 | 46.7 | -0.02 | 46.6 | 47.7 | -0.02 |
| Former smokers, % | 19.3 | 19.0 | 0.008 | 5.6 | 5.6 | -0.002 |
| **Clinical history of CHD** |  |  |  |  |  |  |
| Prior angina pectoris, % | 9.6 | 9.9 | -0.01 | 11.2 | 10.5 | 0.02 |
| Prior MI, % | 8.6 | 8.7 | -0.005 | 11.1 | 10.6 | 0.02 |
| Prior PCI, % | 5.5 | 5.4 | 0.002 | 12.1 | 11.9 | 0.005 |
| Prior CABG, % | 1.3 | 1.4 | -0.002 | 0.8 | 0.8 | 0.004 |
| **Clinical history of CVD** |  |  |  |  |  |  |
| Peripheral arterial disease, % | 2.8 | 2.8 | -0.002 | 1.5 | 1.4 | 0.004 |
| **Clinical presentation on admission** | |  |  |  |  |  |
| Systolic blood pressure, mmHg | 134.2 ± 28.5 | 134.8 ± 26.2 | -0.02 | 138.4 ± 29.1 | 138.5 ± 27.3 | -0.004 |
| Heart rate, bpm | 77.6 ± 19.1 | 78.4 ± 18.9 | -0.04 | 80.2 ± 18.5 | 80.6 ± 18.1 | -0.02 |
| Serum creatinine levels, mg/dL | 1.0 ± 0.5 | 1.0 ± 0.5 | -0.03 | 1.1 ± 0.7 | 1.0 ± 0.7 | 0.03 |
| **Outcomes** |  |  | ***p* value** |  |  | ***p* value** |
| 30-day mortality, % | 5.6 | 2.6 | 0.0005 | 8.0 | 4.1 | <0.0001 |
| Risk Ratio (95% CI) | 2.17 (1.48 – 3.18) | | 0.0001 | 2.05 (1.68 – 2.50) | | <0.0001 |
| Data are presented as percentages (%) or mean ± standard deviation, unless otherwise specified.  Abbreviations: bpm=beats per minute; CABG=coronary artery bypass graft; CHD=coronary heart disease; CVD=cardiovascular disease; PAD=peripheral artery disease; PCI=percutaneous coronary intervention; STEMI=ST-segment elevation myocardial infarction | | | | | | |

| **Table S17.** Interaction test: calculations for comparing two estimated risk ratios (women vs men) for 30-day mortality by inverse probability weighting: high-income countries versus middle-income countries in STEMI patients undergoing revascularization | | | |
| --- | --- | --- | --- |
|  |  | **Group 1**  **[High-income countries]**  **(n = 8,466)** | **Group 2**  **[Middle-income countries]**  **(n = 13,620)** |
| **1** | **RR** | 2.17 | 2.05 |
| **2** | **log RR** | 0.77 | 0.72 |
| **3** | **95% CI for RR** | 1.48 – 3.18 | 1.68 – 2.50 |
| **4** | **95% CI for log RR** | 0.39-1.16 | 0.52-0.92 |
| **5** | **Width of CI** | 0.76 | 0.40 |
| **6** | **SE (=width / (2*1.96))** | 0.20 | 0.10 |
| **Difference between log risk ratios** | | | |
| **7** | **d (=**$\boldsymbol{E}_{\boldsymbol{1}}\boldsymbol{-}\boldsymbol{E}_{\boldsymbol{2}}$**)** | **0.06** | |
| **8** | **SE (d)** | **0.22** | |
| **9** | **CI (d)** | 0.49 | |
| **10** | **Test of Interaction** | 0.26 (***p* value: 0.40**) | |
| **Ratio of risk ratios** | | | |
| **11** | **RRR ( =exp(d) )** | 1.06 | |
| **12** | **CI (RRR)** | 0.69-1.63 | |

| **Table S18.** Inverse probability weighting: clinical factors and outcomes stratified by sex and economic status in NSTEMI patients undergoing revascularization | | | | | | |
| --- | --- | --- | --- | --- | --- | --- |
|  | **High income countries** | | | **Middle income countries** | | |
| **Characteristics** | **Women**  **(n=500)** | **Men**  **(n=1,286)** | **Standardized difference** | **Women**  **(n=699)** | **Men**  **(n=1,790)** | **Standardized difference** |
| Age, years | 65.2 ± 11.3 | 65.3 ± 11.6 | -0.01 | 60.2 ± 11.4 | 60.9 ± 11.0 | -0.06 |
| **Cardiovascular risk factors** |  |  |  |  |  |  |
| Diabetes, % | 22.5 | 22.2 | 0.007 | 26.3 | 26.9 | -0.01 |
| Hypertension, % | 65.8 | 66.4 | -0.01 | 71.5 | 72.2 | -0.02 |
| Hypercholesterolemia, % | 49.3 | 50.0 | -0.01 | 37.8 | 38.5 | -0.01 |
| Current smokers, % | 30.1 | 30.8 | -0.02 | 41.9 | 40.8 | 0.02 |
| Former smokers, % | 29.7 | 28.4 | 0.03 | 4.9 | 4.4 | 0.03 |
| **Clinical history of CHD** |  |  |  |  |  |  |
| Prior angina pectoris, % | 21.7 | 20.6 | 0.03 | 12.6 | 13.6 | -0.03 |
| Prior MI, % | 20.5 | 19.9 | 0.01 | 19.5 | 19.9 | -0.01 |
| Prior PCI, % | 15.7 | 14.8 | 0.02 | 11.4 | 10.8 | 0.02 |
| Prior CABG, % | 5.9 | 5.9 | -0.001 | 2.1 | 1.9 | 0.02 |
| **Clinical history of CVD** |  |  |  |  |  |  |
| Peripheral arterial disease, % | 4.9 | 4.7 | 0.007 | 0.8 | 0.8 | 0.03 |
| **Clinical presentation on admission** |  |  |  |  |  |  |
| Systolic blood pressure, mm Hg | 144.1 ± 26.5 | 144.3 ± 47.2 | -0.004 | 147.1 ± 26.3 | 146.5 ± 25.8 | 0.02 |
| Heart rate, bpm | 77.2 ± 18.4 | 77.3 ± 18.0 | -0.002 | 81.8 ± 17.9 | 81.9 ± 16.9 | -0.006 |
| Serum creatinine levels, mg/dL | 1.2 ± 1.2 | 1.1 ± 0.9 | 0.05 | 1.0 ± 0.4 | 1.0 ± 0.5 | 0.01 |
| **Outcomes** |  |  | ***p* value** |  |  | ***p* value** |
| 30-day mortality, % | 2.3 | 1.6 | 0.37 | 3.2 | 2.8 | 0.55 |
| Risk Ratio (95% CI) | 1.43 (0.69 – 2.96) | | 0.34 | 1.17 (0.71 – 1.94) | | 0.54 |
| Data are presented as percentages (%) or mean ± standard deviation, unless otherwise specified.  Abbreviations: bpm=beats per minute; CABG=coronary artery bypass graft; CHD=coronary heart disease; CVD=cardiovascular disease; NSTEMI=non-ST-segment elevation myocardial infarction; PAD=peripheral artery disease; PCI=percutaneous coronary intervention | | | | | | |

| **Table S19.** Interaction test: calculations for comparing two estimated risk ratios (women vs men) for 30-day mortality by inverse probability weighting: high-income countries versus middle-income countries in NSTEMI patients undergoing revascularization | | | |
| --- | --- | --- | --- |
|  |  | **Group 1**  **[High-income countries]**  **(n = 8,466)** | **Group 2**  **[Middle-income countries]**  **(n = 13,620)** |
| **1** | **RR** | 1.43 | 1.17 |
| **2** | **log RR** | 0.36 | 0.16 |
| **3** | **95% CI for RR** | 0.69 – 2.96 | 0.71 – 1.94 |
| **4** | **95% CI for log RR** | -0.37-1.09 | -0.34-0.66 |
| **5** | **Width of CI** | 1.46 | 1.01 |
| **6** | **SE (=width / (2*1.96))** | 0.37 | 0.26 |
|  | | | |
| **7** | **d (=**$\boldsymbol{E}_{\boldsymbol{1}}\boldsymbol{-}\boldsymbol{E}_{\boldsymbol{2}}$**)** | **0.20** | |
| **8** | **SE (d)** | **0.45** | |
| **9** | **CI (d)** | -0.68-1.09 | |
| **10** | **Test of Interaction** | 0.44 **(*p* value: 0.33)** | |
|  | | | |
| **11** | **RRR (=exp(d) )** | 1.22 | |
| **12** | **CI (RRR)** | 0.50-2.96 | |

#

# **REFERENCES**

1. Bugiardini R, Badimon L**.** The International Survey of Acute Coronary Syndromes in Transitional Countries (ISACS-TC): 2010-2015. Int J Cardiol. 2016;217 Suppl:S1-6.
2. Alabas OA, West RM, Gillott RG, Khatib R, Hall AS, Gale CP**.** Evaluation of the Methods and Management of Acute Coronary Events (EMMACE)-3: protocol for a longitudinal study. *BMJ Open*. 2015;**5**(6):e006256.
3. Schwartz GG, Steg PG, Szarek M, et al. Alirocumab and cardiovascular outcomes after acute coronary syndrome. *N Engl J Med* 2018;**379**:2097-107. DOI: 10.1056/NEJMoa1801174
4. National Health Interview Survey- Adult Tobacco Use Information. 2017. <https://www.cdc.gov/nchs/nhis/tobacco/tobacco_glossary.htm>.
5. van Buuren, S, Groothuis-Oudshoorn K. "mice: Multivariate imputation by chained equations in R." *Journal of Statistical Software*. 2011;**45**(3). doi:10.18637/jss.v045.i03
6. He Y. Missing data analysis using multiple imputation: getting to the heart of the matter. *Circ Cardiovasc Qual Outcomes*. 2010;**3**(1):98-105.
7. Austin PC, Stuart EA. Moving towards best practice when using inverse probability of treatment weighting (IPTW) using the propensity score to estimate causal treatment effects in observational studies. *Stat Med* 2015; **34**(28): 3661-79.
8. Katz D, Baptista J, Azen SP, et al. Obtaining Confidence Intervals for the Risk Ratio in Cohort Studies. *Biometrics* 1978;**34**(3):469-74. doi: 10.2307/2530610
9. Dongsheng Y, Dalton JE. A unified approach to measuring the effect size between two groups using SAS®. SAS Global Forum. Vol. 335. 2012.
10. Altman DG, Bland JM. Interaction revisited: the difference between two estimates. *BMJ* 2003; **326**(7382): 219.
